# Huntingtin knockdown dysregulates autophagic degradation of Apolipoprotein E

**DOI:** 10.1177/18796397251391110

**Published:** 2025-10-29

**Authors:** Gianna M Fote, Nicolette R McClure, Robert M Bragg, Jharrayne McKnight, Leslie M Thompson, Jeffrey B Carroll, Joan S Steffan

**Affiliations:** 1 Department of Biological Chemistry, University of California Irvine, Irvine, CA, USA; 2 Department of Neurological Surgery, University of California Irvine, Irvine, CA, USA; 3 Department of Neurobiology and Behavior, University of California Irvine, Irvine, CA, USA; 4 Department of Neurology, 7284 University of Washington, Seattle, WA, USA; 5 Department of Psychiatry and Human Behavior, University of California Irvine, Irvine, CA, USA; 6 Institute for Memory Impairments and Neurological Disorders, University of California Irvine, Irvine, CA, USA; 7 Center for Epigenetics and Metabolism, University of California Irvine, Irvine, CA, USA

**Keywords:** apolipoprotein E, Huntingtin, chaperone-mediated autophagy, macroautophagy, Huntington's disease, liver

## Abstract

**Background:**

The HTT protein, mutated in Huntington's disease, is expressed throughout the body, and loss of HTT function as an autophagic scaffold may affect tissues and cellular processes. These processes include lipid metabolism potentially regulated upstream by Apolipoprotein E (APOE) and clearance of APOE itself.

**Objective:**

To determine the impact of HTT reduction on autophagy and clearance of APOE in cell culture and in mouse liver *in vivo*.

**Methods:**

Western blot analysis was performed on liver tissue from tamoxifen-treated mice with and without UBC-Cre expression, required for tamoxifen-induced HTT knockout (KO). siRNA was used to knockdown (KD) HTT in HepG2 immortalized liver cells.

**Results:**

HTT KO in mouse liver reduces levels of LAMP2A, a protein essential for chaperone-mediated autophagy (CMA) which we previously found is required for optimal degradation of APOE and HTT in cultured cells. In turn, APOE levels were increased with HTT KO in mouse liver, while HTT KD in cell culture decreased levels of APOE.

**Conclusions:**

In the context of liver tissue, reduced CMA may contribute to accumulation of APOE and autophagic cargo resulting from a loss of HTT function in autophagy. The extent to which macroautophagy is upregulated to cope with reduced CMA found with HTT KO may be tissue specific, which may relate to the selectivity of tissue pathogenesis observed in Huntington's disease where loss of normal HTT function may be involved. This study may help elucidate the consequences of systemic HTT reduction on autophagy in liver tissue.

## Introduction

The Huntingtin protein (HTT), which when mutated causes Huntington's disease (HD), is expressed in tissues throughout the body.^1^ HTT has important functions including those in vesicle transport^2–4^ and as an autophagic scaffold.^5–7^ Autophagy is the process of transporting cellular contents to the lysosome for degradation; HTT knockout, mutant HTT or loss-of-function models each show an accumulation of autophagy receptor p62, ubiquitin puncta, lipids and empty vesicles.^5–8^ HTT has been found by several laboratories to interact directly with p62, and recently we found that HTT itself contains an ubiquitin-binding domain and regulates autophagic targeting of mitochondrial and RNA-binding proteins.^5–7^^,9^ Many different autophagic degradation pathways have been described that are important for cellular homeostasis.^10^ The kinds of autophagy HTT may scaffold are still under investigation. The C-terminal domain of HTT has similarity to yeast Atg11, an autophagic scaffold involved in both macroautophagy and microautophagy, relevant to the yeast cytoplasm-to-vacuole targeting (Cvt) pathway, nucleophagy and mitophagy.^6,11^ In mammalian cells, HTT has been suggested to function in basal macroautophagy, lipophagy, aggrephagy, mitophagy, nucleophagy and other forms of selective autophagy; a reduction in HTT function with HTT knockout (KO) or mutation may be compensated for by an upregulation of HTT-independent starvation-induced macroautophagy.^3^^,^^5–7^^,9,11,12^ Although it is known that HTT is required for neurodevelopment and neurological function,^4,13,14^ it is not completely understood whether normal HTT function is required in adult peripheral tissues.^1^ The HD CAG repeat expansion not only causes a gain of aberrant function, but appears to also reduce normal HTT functions.^4^ Peripheral HD symptoms include global metabolic dysfunction, weight loss, dysfunction of highly metabolic tissues such as cardiac and skeletal muscle,^15^ lipid droplet accumulation,^5,8,16^ and liver dysfunction.^17^ Liver tissue is highly autophagically active, and it is possible that wild-type HTT's function as an autophagic scaffold may be important for normal liver function. Selective and non-selective macroautophagy contribute to the liver's ability to respond to stress or starvation.^18,19^ Chaperone mediated autophagy (CMA), a mechanism of direct lysosomal uptake without an autophagosome intermediate, may be scaffolded by HTT^7,11^ and is also critical for normal liver function, contributing to whole-body energetic balance and preventing hepatosteatosis.^20,21^ It is not yet known how HTT lowering may affect autophagic balance in liver tissue *in vivo.* However, there is limited information available regarding the effects of HTT lowering in peripheral tissues, particularly in adult and aged animals. Recently, the Carroll group^22^ investigated the molecular, cellular and metabolic impacts of chronic HTT lowering in hepatocytes from viable and healthy mice with hepatocyte-specific *Htt* KO (*Htt^LKO/LKO^*). In this study, increased circulating bile acids, cholesterol and urea, hypoglycemia, impaired adhesion and alterations in liver zonation at the transcriptional, histological and plasma metabolite levels consistent with impaired beta-catenin function were observed. This work described an unexpected role for wild-type HTT function in cellular fate and liver zonation in mice that develop in the absence of hepatocyte HTT and emphasized an important role for wild-type HTT in normal liver function.^22^ Follow-up whole body lowering global HTT KO studies using two separate tamoxifen-inducible Cre lines showed that a loss of HTT at 2 months of age leads to progressive tremors and severe subcortical brain calcification together with sustained induction of circulating neurofilament light chain.^23^

Apolipoprotein E (APOE) is a lipid-carrying protein expressed primarily in brain and liver that has significantly reduced expression in models with mHTT expression.^24,25^ In humans (but not mice or other mammals) there are three isoforms of APOE (APOE2, APOE3 and APOE4); APOE4 allele is the greatest genetic risk factor for late onset Alzheimer's disease (AD), as well as atherosclerosis and diabetes.^26–29^ In neuronal cells, APOE4 exerts a gain-of-toxicity by alkalinizing the lysosomal lumen, impairing protein degradation.^30^ In cell culture, we showed that APOE4 dysregulates autophagy and accumulates in enlarged endosomes, alters autophagic flux, and changes proteomic contents of lysosomes following internalization, which may contribute to AD pathogenesis.^31^

Although there is evidence that lipid metabolism involving APOE and liver function are dysregulated in HD or with mHTT expression, the role of HTT in these processes has not been fully elucidated. Brain cholesterol synthesis and metabolism are reduced in HD^24,32^ and lipid droplets accumulate due to increased lipid uptake and deficits in lipophagy.^5,8,16^ In human iPSC-derived medium spiny neurons expressing mutant HTT, APOE is dysregulated, and the size of lipid droplets is increased and their turnover impaired, similar to what has been observed in APOE4 or APOE knockdown astrocytes.^16,33^ Hepatic mitochondrial dysfunction has been observed in HD,^34^ and levels of alkaline phosphatase, gamma-glutamyl transferase, total cholesterol and blood glucose are elevated in patients with manifest HD, a reflection of altered liver function.^17^ Liver tissue is highly active in lipid synthesis and metabolism,^35^ but liver lipid homeostasis *in vivo* has been largely unexplored in the context of HTT knockdown. We therefore investigated levels of key autophagy proteins after HTT knockdown *in vivo* in liver tissue and in cell culture. Here, we find that APOE interacts with HTT and has weak sequence similarity to the yeast autophagy receptor Atg39 that interacts directly with yeast Atg11, similar to the C-terminal domain of HTT,^6,11^ suggesting that HTT may function together with APOE in a form of selective autophagy. Induced adult-onset HTT silencing in mice was investigated to determine effects on wild-type mouse liver levels of LAMP2A important for CMA, autophagic receptor protein p62, the LC3II/I ratio, which is an assay for increased macroautophagy, and APOE. APOE levels were found to be significantly increased in HTT KO liver, while LAMP2A levels were reduced, suggesting impaired CMA function *in vivo* in liver with HTT KO, and a possible role for HTT and APOE in CMA in liver.

## Methods

### Mouse work

#### Mice

We used a subset of the conditional *Htt*-knockout mice from a previously described cohort.^23^ Briefly, mice were generated by crossing a tamoxifen-inducible cre recombinase allele (UBC-cre; JAX stock 007001) with a *Htt* flox allele,^36^ both on a consistent C57Bl6/J background. Mice were maintained at homozygosity for the *Htt* flox allele and hemiozygosity for the cre allele. All mice were bred at the McLaughlin Research Institute (MRI) and housed in cages of 3–5 mice with access to food and water ad libitum, with lights maintained on a 12-h light/dark cycle. The MRI institutional animal care and use committee approved all procedures under protocol 2020-JC-29.

#### Mice, treatment groups, and tissue collection

For the analyses described here, we used whole body lowering tamoxifen-treated homozygous *Htt* flox with UBC-Cre (Hereafter referred to as KO) and tamoxifen-treated littermates without UBC-Cre as a control (Hereafter referred to as Controls). Mice were treated with Tamoxifen (Sigma T5648) via I.P. injection at 75 mg/kg once daily for 5 consecutive days at 2 months of age. Mice were sacrificed at 14 months of age and the caudate liver lobe was collected for analyses.

#### Western blot analysis of liver tissue

Liver tissue was broken in modified RIPA buffer containing: Tris-HCl: 50 mM, pH 7.4, NP-40: 1%, Na-deoxycholate: 0.25%, NaCl: 150 mM, EDTA: 1 mM, Pierce mini protease inhibitor pellet (Fisher Scientific A32953). Samples were homogenized in a Potter-Elvehjem style tissue homogenizer, sonicated, then centrifuged for 15 min at 4 degrees centigrade for 20,000 rcf to remove the fibrous tissue, and supernatant analyzed by Bradford assay. 10–50 μg of protein was then used for SDS/PAGE. 4–12% Bolt Bis-Tris Plus Mini Protein Gels (NW04125BOX) were used with either MOPS or MES buffers (Invitrogen NP0001 and NP0002 respectively); NuPage 3–8% Tris-Acetate Mini Protein Gels (Thermo Fisher Scientific EA03785BOX) were used with Tris-acetate running buffer (Fisher Scientific LA0041). Gels were then transferred onto 0.2 μ nitrocellulose (BioRad). Whole protein was quantified using Revert Total Protein Stain assay (LICOR Biosciences 926-11016), and the membrane was blocked with Intercept (TBS) Blocking Buffer (LICOR biosciences 927-60010) for 1 h. The membrane was then incubated in primary antibody overnight, washed three times with TBS-0.1% Tween-20, and incubated for 1 h in near-infrared-conjugated secondary antibody (LICOR biotech IRDye) in intercept block supplemented with 0.1% Tween-20. Membranes were imaged on a LICOR scanner and quantified using Empiria Software.

### Cell culture

Human cell lines HepG2, HeLa and HEK293 cells were maintained in DMEM (Corning 10-017-CV) supplemented with 10% FBS (ThermoFisher 26140079). Cells were cultured at 37°C, 5% CO_2_. HepG2 expresses APOE3, while HeLa and HEK293 do not express detectable APOE.

### Western blot of cell culture lysates

Cells were harvested using lysis buffer containing 10% glycerol, 20 mM Tris pH 7.5, 137 mM NaCl, 1% NP40, 5 mM EDTA, phosphatase inhibitors 2 (Millipore Sigma, P5726) (1:1000) and 3 (Millipore Sigma P0044) (1:1000), 5 mM nicotinamide (Sigma 72340), 5 mM butyric acid, 1 mM PMSF, 10 μg/mL aprotinin (Sigma-Aldrich A1153), 10 μg/mL leupeptin (Sigma-Aldrich L2884), and one Pierce mini protease pellet (Fisher Scientific A32953) per 10 mL of lysis buffer. Lysates were sonicated (3 times for 10 s at 40% power) and 20 μg of protein was then used for SDS/PAGE and western analysis as described above using liver tissue, however in this case gels were transferred onto Immobilon-FL PVDF (Millipore Sigma IPFL00010).

### Co-immunoprecipitation

For Figure 5(a), 1 μL of anti-HTT 5526 antibody was pre-incubated with 500 μg lysate for 1 h. Dynabeads M-280 anti-rabbit (ThermoFisher 11203D) beads were pre-blocked with lysis buffer with no glycerol and with 0.1% BSA for 30 min. Blocked beads and lysate-antibody mixture were combined and incubated at 4°C for 1 h. Beads were then washed for 5 min twice in buffer with BSA and once without BSA and analyzed by western blot. Western blot was performed as described.^31^ For Figure 5(b), cells were lysed and Myc-tagged protein was immunoprecipitated as previously described.^6^

### siRNA

siRNA was reverse-transfected using RNAiMAX transfection reagent (ThermoFisher 13778100) using a protocol supplied by the manufacturer. Media was changed the following day, and cells were harvested 48 h after transfection. siHTT siRNA sequence (sense): GGUUUAUGAACUGACGUUAUU. siCTRL sequence (sense): GCGAACGACUUACGCGUUUAUU. The target is within the HTT gene coding region between amino acids 357-362 VYELTL.

### APOE3 secretion

HepG2 cells expressing and secreting APOE3 were plated and reverse-transfected with control or HTT siRNAs in Corning 96 well plates. 48 h later, 15 microliters of conditioned media was collected from HepG2 cells and analyzed by western blot.

### Endocytosis live-cell imaging assay

HepG2 cells were plated and reverse-transfected with control of HTT siRNAs in Corning 96 well plates. Forty-eight hours later, media was replaced with 50% fresh media, 50% media conditioned by HEK293 cells stably expressing and secreting APOE3-mCh,^31^ or with fresh media containing LDL-pHrodo (Thermo Fisher Scientific L3455) at a concentration of 10 µg/mL. Fluorescence and phase images were acquired using an IncuCyte Live Cell Analysis System (Sartorius) with a 20x lens, with n = 6 wells per group, 3 images per well. Mask settings were created using the IncuCyte S3 software and applied to all images for a particular experiment.

### Autophagy inhibition

HepG2 cells reverse transfected with siRNA were treated with Bafilomycin A1 (Cayman Chemical 11038) (5 0 nM) for 4 h before lysis for western blot analysis.

### LysoIP protocol

HepG2 cells stably expressing TMEM192-HA^31^ were reverse transfected with siHTT or siCTRL in 15 cm plates, with 3 replicate plates per siRNA. Media was changed 24 h after plating. Forty-eight hours after transfection cells were then incubated with 50 nmol Baf for 4 h and lysed using a dounce homogenizer. LysoIP was then performed as previously described.^37^

### Quantitative RT-PCR

cDNA synthesis and qPCR analysis was performed as described.^31^ Cells were harvested and snap frozen. Cells were resuspended in RLT buffer from Qiagen, and RNA was extracted using the RNeasy mini kit (Qiagen 74106). One microgram of RNA was reverse-transcribed into cDNA using qScript (VWR 101414-106). RT-qPCR was performed using SYBR green supermix (Biorad 1725124). Transcript levels were normalized to RPLPO Ct values. Primers: Human RPLPO (F) TGGTCATCCAGCAGGTGTTCGA (R) ACAGACACTGGCAACATTGCGG, Human APOE (F) GGGTCGCTTTTGGGATTACCTG (R) CAACTCCTTCATGGTCTCGTCC.

### Incucyte live-cell imaging

HepG2 (250,000 cells/mL) were plated into Corning 96 well plates. Images were acquired in the plane of highest contrast using an IncuCyte Live Cell Analysis System (Sartorius). Three images per well were collected in phase-contrast, green and red fluorescence with a 20x lens, with n = 3 wells per group. Quantitative fluorescence analysis was performed using the IncuCyte S3 software by creating mask settings that were applied to all images for a particular experiment.

### Statistics

All cell culture experiments were performed twice with at least 3 biological triplicates (3 separate cell culture wells). Statistics were performed using PRISM software. Error bars represent standard error of the mean. Two-way or one-way ANOVA with multiple comparisons was corrected using Tukey's correction: p < 0.05 *, p < 0.01 **, p < 0.001 ***, p < 0.0001 ****. Pairwise statistical analysis was achieved by unpaired-T test. Significant p-values are indicated as follows: p < 0.05 *, p < 0.01 **, p < 0.001 ***, p < 0.0001 ****.

### Antibodies

**Table:** 

Target	Company and Catalogue number	Application, concentration
APOE	Biolegend 803301	Western blot 1:500
mCherry	Biolegend 677702	Western blot 1:500
P62/SQSTM1	Abnova PAB16850	Western blot 1:1000
Mouse LAMP2A	ThermoFisher 51-2200	Western blot 1:1000
Mouse APOE	Abcam ab183596	Western blot 1:1000
Human APOE	Invitrogen 701241	Western blot 1:500
LC3A/B	Cell Signaling	Western blot 1:1000
Huntingtin	Abcam EPR5526	Western blot 1:1000
α-tubulin	Sigma T6074-200	Western blot 1:2000
Human LDLR	MyBioSource MBS9608278	Western blot 1:1000
Human LRP1	Biolegend 858702	Western blot 1:1000
Human LAMP1	Abcam ab24170	Western blot 1:1000
GFP tag	Takara 632569	Western blot 1:1000
Myc tag	Sigma Aldrich 9E10 clone	Western blot 1:1000
Actin	Sigma Aldrich A2066	Western blot 1:1000
Secondary ms HRP	Jackson Immuno Research 115035146	Western blot 1:20000
Secondary NIR 800 ms	LICOR 92632210	Western blot 1:20000
Secondary Nir 700 Rb	Licor 92632211	Western Blot 1:20000

## Results

### Levels of autophagy proteins and APOE are altered in HTT KO liver tissue

**Figure 1.**
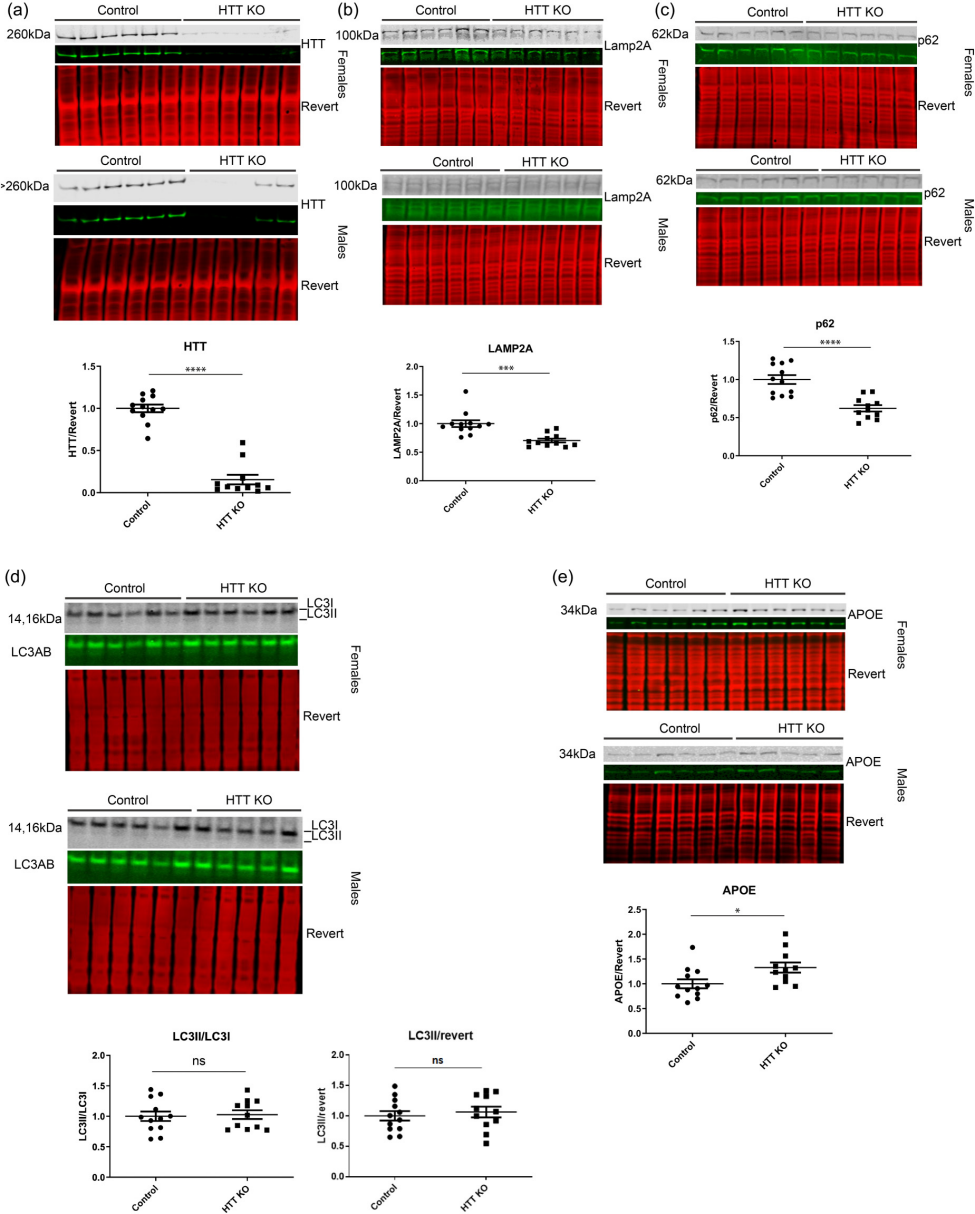
HTT KO in mouse liver dysregulates autophagy proteins. Western blot analysis was performed on liver tissue from male and female tamoxifen-treated mice with and without UBC-Cre expression required for tamoxifen-induced HTT knockout (KO).^23^ (a) HTT was significantly knocked out in female and male mice expressing UBC-Cre, although the females had the most consistent KO, as two males had some expression but it was still significantly lowered. (b, c) LAMP2A and p62 levels were significantly reduced in HTT KO mouse liver. (d, e) The LC3II/I ratio and LC3II/total protein stain, revert, was not significantly altered, and APOE levels were significantly increased in HTT KO mouse liver. For all blots, protein of interest is shown in both pseudo-color and gray scale. Relative abundance of proteins per revert total protein stain was normalized to wt controls and graphed.

In order to assess whether dysregulation of autophagy is associated with the HTT reduction in mouse liver, western blot analysis was performed on liver tissue obtained from male and female tamoxifen-treated mice with and without UBC-Cre expression required for global tamoxifen-induced HTT knockout (KO) (Figure 1(a)).^23^ Levels of the CMA receptor LAMP2A were significantly reduced by HTT KO (Figure 1(b)), consistent with an inhibition of CMA with reduced HTT function. In HTT KO animals, the levels of selective autophagic receptor protein p62 were significantly reduced (Figure 1(c)). The ratio of lipidated LC3 (LC3II), which is conjugated to autophagosomes relative to unlipidated LC3I, can serve as a marker of macroautophagic flux. The ratio of LC3II/I was unchanged (Figure 1(d)) suggesting macroautophagy was not affected in this context. Levels of LC3II, normalized to total protein revert stain, were also not significantly altered with HTT KD. APOE, which can be cleared by both CMA and macroautophagy,^31^ was significantly elevated with HTT KO in liver (Figure 1(e)). For Figure 1, the numbers were normalized to the average of the control for each sex, then averaged together to generate one graph including both sexes pooled.

### Macroautophagy is upregulated and APOE degradation is enhanced with HTT KD in cultured cells

**Figure 2.**
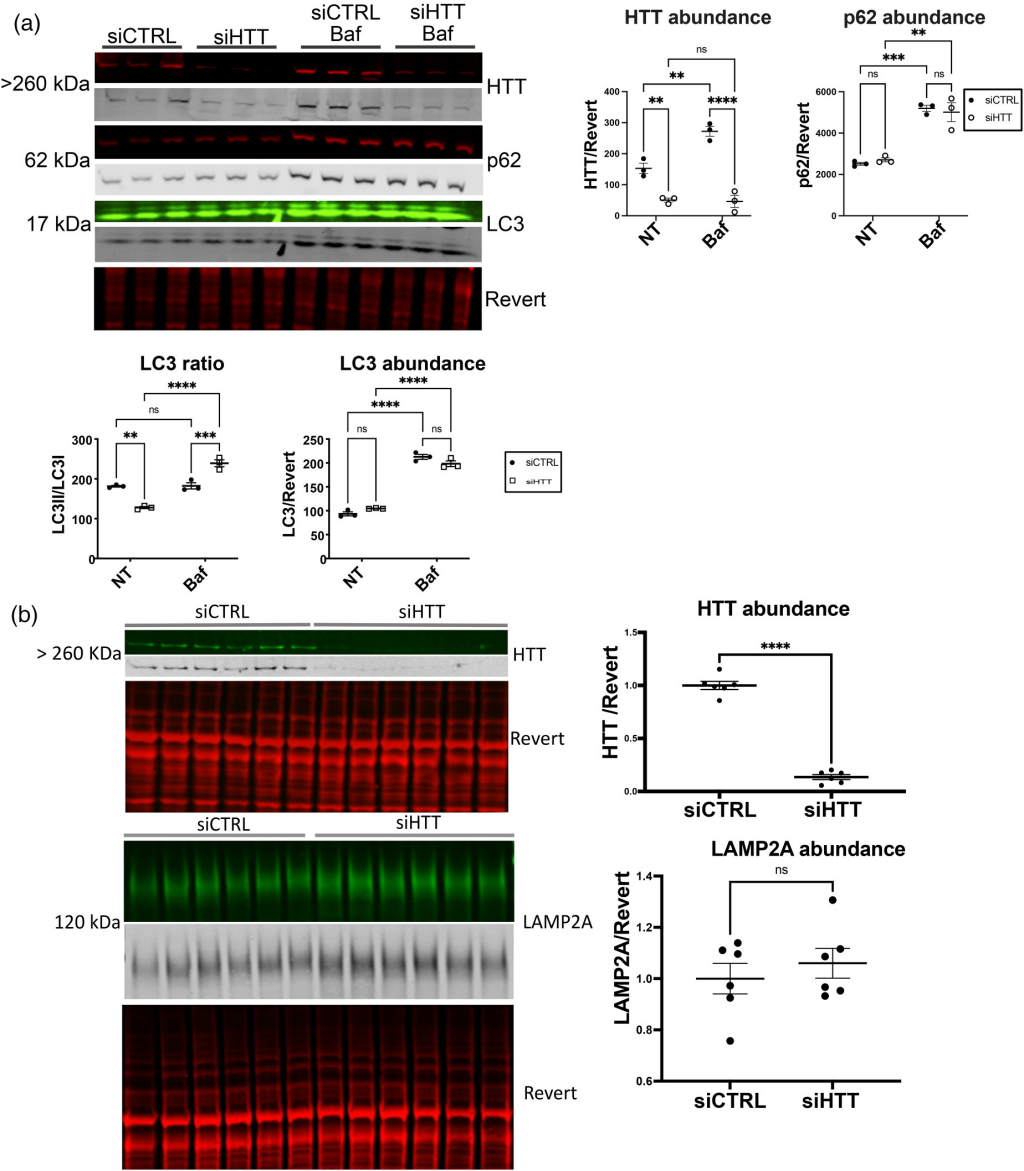
HTT KD in HepG2 cells. siRNA against human HTT was transfected into HepG2 cells. Cells were harvested 48 h later, and 20μg of lysate was analyzed by western blot. Western blots were probed with anti-HTT, anti-p62, anti-LC3 or anti-LAMP2A, scanned using LICOR and bands were quantified using Empiria software relative to revert loading control. (a) Both HTT and the LC3II/I ratio were significantly reduced with HTT KD in HepG2, while p62 levels were unaltered. Bafilomycin (Baf) treatment significantly increased levels of HTT, p62 and LC3. (b) Levels of LAMP2A were not significantly altered in HepG2 cells with HTT knockdown. For all blots, protein of interest is shown in both pseudo-color and gray scale.

Given the observed reduction of LAMP2A levels in liver tissue with HTT KO, consistent with reduced CMA,^38^ we further investigated the levels of autophagy proteins using western analysis with HTT knockdown (KD) *in vitro*. To focus on liver systems, human immortalized hepatic (HepG2) cells were transfected with siRNA against HTT, and significant HTT KD was achieved (Figure 2(a) and (b)). Treatment with Bafilomycin A1 (Baf) blocks lysosomal degradation of autophagosomes, and significantly increased HepG2 levels of p62. HTT KD reduced the LC3II/LC3I ratio, demonstrating increased degradation of autophagosomes, whereas HTT KD cells treated with Baf have an increased ratio, suggesting accumulation of autophagosomes with lysosome blockade (Figure 2(a)). Thus, HTT KD may increase macroautophagic flux in these cells, which we did not observe *in vivo* in liver, Figure 1(d). In cell culture, Figure 2(a), macroautophagy is robustly activated in HepG2 cells, with LC3II being the predominant species of LC3. While HepG2 cancer cells are transformed liver cells, the level of basal macroautophagy is very much higher than what we observed in liver tissue, as shown in Figure 1(d) vs. Figure 2(a), independent of HTT abundance. Levels of LAMP2A were not significantly altered with HTT KD in HepG2 (Figure 2(b)), unlike the significant reduction we found in liver with HTT KO (Figure 1(b)).

**Figure 3.**
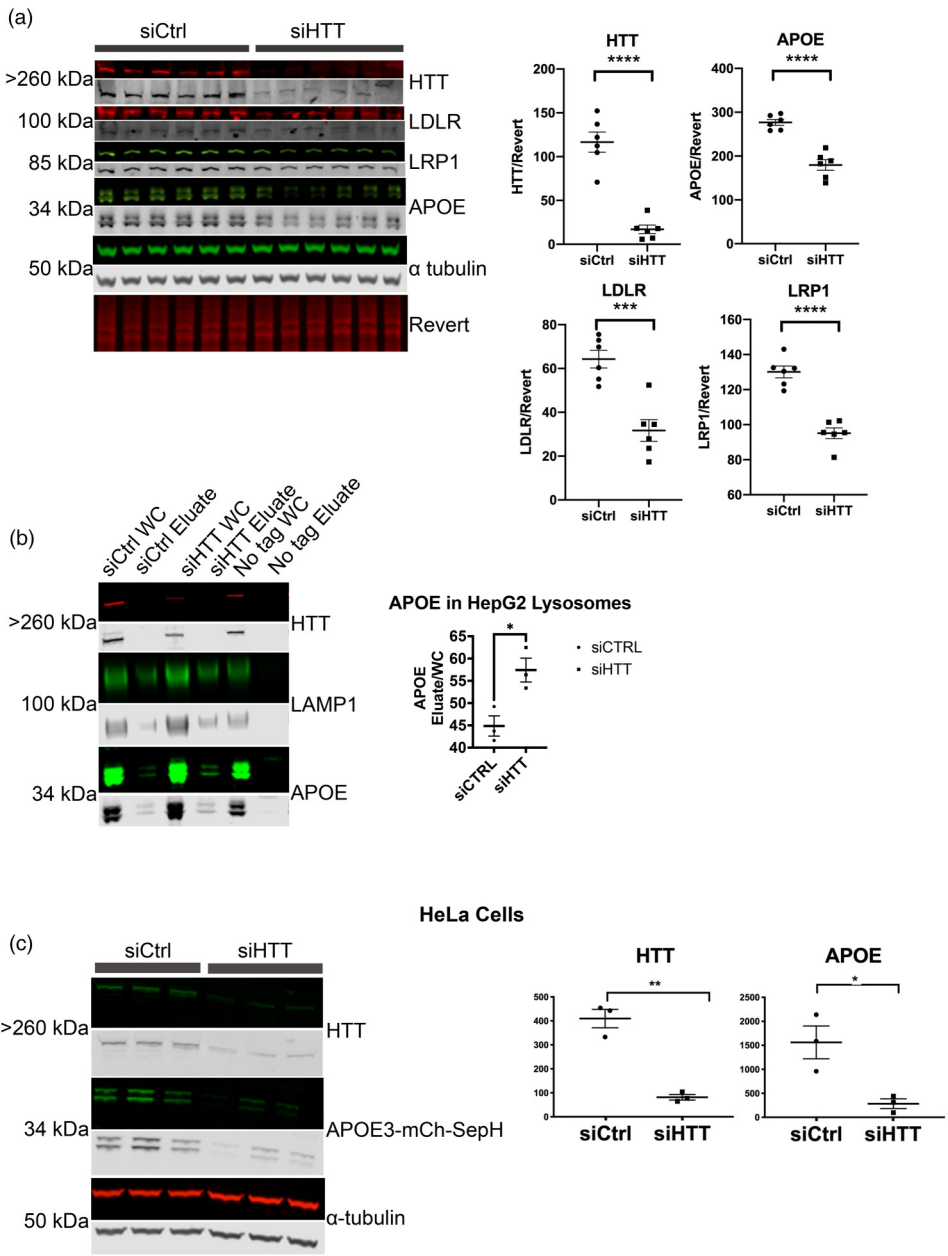
HTT KD in HepG2 cells increases endogenous APOE lysosomal degradation. (a) HTT KD significantly reduces levels of endogenous APOE3, LDLR, LRP1 in HepG2 cells. siRNA against human HTT was transfected into HepG2 cells. Cells were harvested 48 h later, and 20 μg of lysate was analyzed by western blot. Western blot was probed with anti-APOE, anti-HTT, anti-LDLR, anti-LRP1, and anti-alpha-tubulin, scanned using LICOR and bands were quantified using Empiria software relative to revert loading control. Levels of HTT, APOE3, LDLR and LRP1 were significantly reduced with HTT KD. (b) Endogenous lysosomal APOE3 was significantly increased in HepG2 cells with HTT KD. siRNA against human HTT was transfected into HepG2 cells expressing the lysosomal protein TMEM-192 tagged with HA for performing LysoIP. Cells were harvested 48 h later, and LysoIP performed. 5% of whole cell and LysoIP eluate were analyzed by western blot using anti-HTT, anti-LAMP1, and anti-APOE, scanned using LICOR and bands were quantified using Empiria software. (c) Transiently transfected APOE3-mCherry-SepHluorin levels were significantly reduced with HTT KD in HeLa cells. APOE3 tagged with mCherry-SepHluorin was co-transfected with siHTT or siCtrl into HeLa cells harvested 48 h later and analyzed by western blot. Membranes were probed with GFP antibody. For all blots, protein of interest is shown in both pseudo-color and gray scale.

We previously found that APOE is degraded by both macroautophagy and LAMP2A-dependent CMA in HepG2 cells.^31^ We therefore investigated APOE levels with HTT KD. HTT reduction in HepG2 cells significantly reduced endogenous APOE3 abundance by western analysis (Figure 3(a)), unlike the increase in mouse APOE we observed with HTT KO in liver *in vivo* (Figure 1(e)). KD of HTT in HepG2 cells also produced a significant reduction in the abundance of lipoprotein receptors LRP1 and LDLR (Figure 3(a)). Immunoprecipitation of intact lysosomes (LysoIP) from HTT KD HepG2 cells confirms that endogenous APOE3 levels were targeted to lysosomes of siHTT cells as lysosomal APOE3 levels were significantly increased (Figure 3(b)), consistent with their reduction in cellular extract and macroautophagic degradation. In addition, we also evaluated transiently transfected APOE3-mCherry-SepHluorin levels in HeLa cells, and found them significantly reduced with HTT KD (Figure 3(c)).

**Figure 4.**
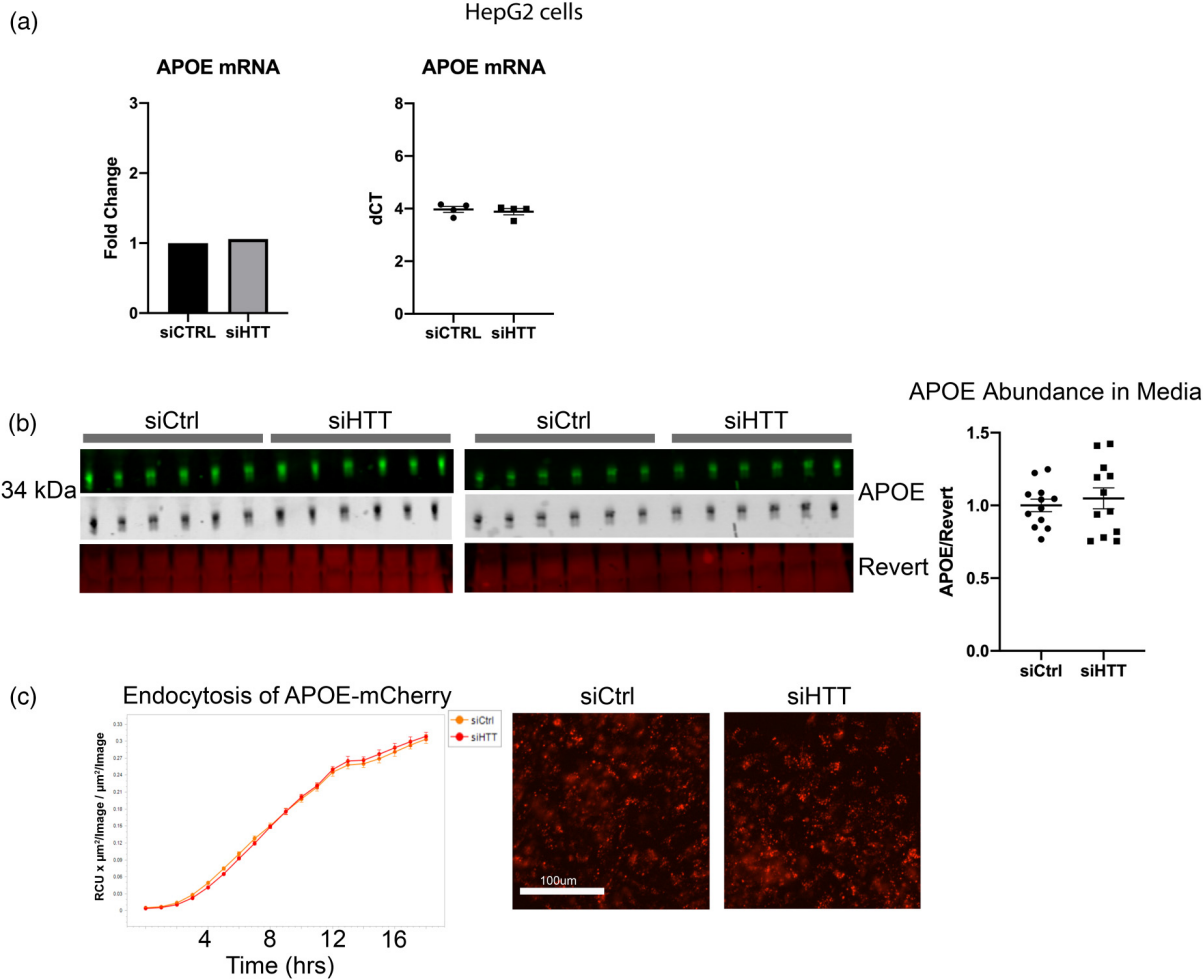
APOE expression, secretion, and uptake are not altered with HTT KD. (a) HTT KD did not alter levels of endogenous APOE transcript in HepG2 cells. qPCR was performed on n = 4 biological replicate wells with technical triplicates of HepG2 cells. (b) No alterations in endogenous APOE3 secretion from HepG2 cells with HTT KD was observed. Media was collected from HepG2 cells transfected with siRNA against HTT or control. Media was analyzed for APOE levels by western blot. (c) APOE3-mCherry uptake by HepG2 cells was not impacted by HTT KD. HepG2 cells were transfected with control or HTT siRNA then were incubated with media from HEK293 cells stably expressing and secreting APOE3-mCherry. Cells were imaged and fluorescence quantified using incucyte to determine possible changes in APOE3-mCherry uptake with HTT KD. For all blots, protein of interest is shown in both pseudo-color and gray scale.

Expression of endogenous APOE3 transcript was unaltered in HepG2 with HTT KD as assessed by quantitative PCR (Figure 4(a)). Considering the reduction in LDLR and LRP1 abundance with HTT KD, we investigated whether the reduction in intracellular APOE3 levels may be due to reduced endocytosis or increased secretion of APOE3. Levels of APOE3 secreted into media by HepG2 cells were not affected by HTT KD (Figure 4(b)). Although HTT KD also significantly reduced abundance of the lipoprotein receptors LRP1 and LDLR (Figure 3(a)), HepG2 endocytosis of fluorescently tagged APOE3 from conditioned media, secreted into media from HEK293 cells stably expressing and secreting APOE3-mCherry, was not affected by HTT KD (Figure 4(c)). In summary, macroautophagy appears to be upregulated in HepG2 cells with HTT KD, which may cause enhanced lysosomal APOE3 degradation in this cancer cell line, while HTT KD does not alter expression, secretion or uptake/internalization of APOE3.

### APOE and HTT interact in transiently transfected cultured cells

**Figure 5.**
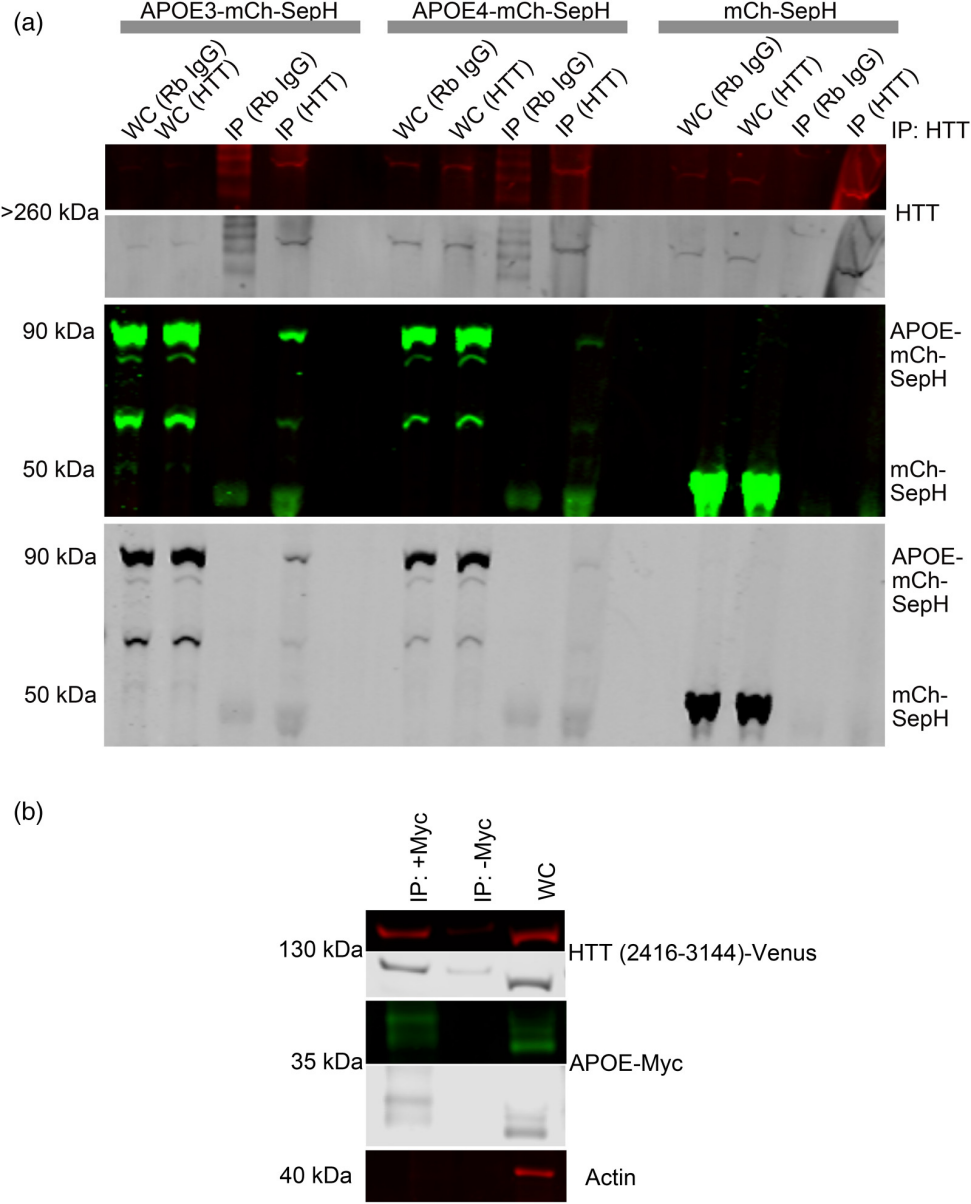
APOE and HTT co-immunoprecipitate from HEK293 cells. (a) APOE3 and APOE4 interact with endogenous full length HTT. APOE3 or APOE4 tagged with mCherry-SepHluorin were transiently transfected into HEK293 cells and co-immunoprecipitated with endogenous HTT protein using anti-HTT antibody 5526. Western blot membranes were probed with anti-GFP antibodies. (b) The receptor-binding domain of human HTT interacts with APOE. APOE-myc and HTT fragment (amino acids 2416-3144) tagged with Venus^6^ were co-transfected into HEK293 cells and then co-immunoprecipitated using APOE-Myc as bait and probing for Venus using GFP antibody. For all blots, protein of interest is shown in both pseudo-color and gray scale.

Since HTT functions as an autophagic scaffold,^5–7^^,11^ and APOE, which is degraded by autophagy^31^ and appears to be modulated by HTT levels, we hypothesized that HTT may interact with APOE. We performed immunoprecipitation of HTT in HEK293 cells stably expressing fluorescent mCherry-SepHluorin tagged APOE3, APOE4, or tag alone (Figure 5(a)). Both APOE3 and APOE4 co-immunoprecipitated with endogenous HTT. The three bands represent modified and unmodified APOE3-mCh-SepH, and APOE3-mCh, and appear to demonstrate a preference of HTT for the modified higher molecular weight species. We previously demonstrated using pharmacologic inhibition that autophagic degradation of APOE in the lysosome requires Golgi trafficking, consistent with preference of HTT as an autophagy protein for the Golgi-modified species.^31^ We then tested the C-terminal Atg11-like fragment of HTT that functions as an autophagy receptor-binding domain (Venus-HTT, HTT amino acids 2416-3144 in pGW1)^6^ and found that it co-immunoprecipitates with APOE3-myc in HEK293 cells (Figure 5(b)). This suggests that APOE and the portion of HTT that scaffolds autophagy receptors may interact directly. Our data using transiently transfected cells overexpressing APOE may support a possible interaction between endogenous APOE and HTT. APOE is a lipid-binding protein that traffics to lipid droplets, modulates their size, and is a lipid droplet surface protein.^33,39^ Atg39, a yeast autophagy receptor that directly interacts with yeast Atg11, was defined in *Saccharomyces cerevisiae* in 2015.^40,41^ Atg39, functioning in an Atg7-independent form of microlipophagy, is involved in the clearance of ubiquitinated proteins associated with lipid droplets.^41^ We compared Atg39 and APOE and found weak amino acid sequence similarity between the two (Supplemental Figure 1), consistent with a potential function for APOE as an Atg7-independent microlipophagy receptor scaffolded by HTT in mammalian cells, functionally similar to yeast Atg39.

## Discussion

In this study we investigated whether HTT is involved in the regulation of autophagic APOE clearance in mouse liver tissue *in vivo* and in immortalized cultured cells *in vitro* by knocking down wild-type HTT protein levels in these systems. We previously found that the APOE protein is cleared primarily by CMA but may also be degraded by macroautophagy in cell culture.^31^ Using western analysis, we now find that adult-onset HTT KO in mouse liver reduces the levels of the CMA receptor protein LAMP2A and increases levels of APOE, which may reflect a reduction in CMA *in vivo* with HTT liver KO. Using the LC3II/I ratio and levels of LC3II/total protein levels in lysate, we determined that macroautophagy was not activated with HTT KO in liver. This is consistent with previous reports that in aged rodent liver, macroautophagy is suppressed as measured by LC3B-II and p62 levels and reduced number of autophagosomes on TEM.^42^ Thus, with no alternative mode of autophagic degradation, loss of HTT in liver *in vivo* may have produced an increase in APOE levels. Alternatively, in HepG2 cultured liver cells, LAMP2A levels were unaltered with HTT KD, while levels of macroautophagy were upregulated and APOE levels were reduced. In cancer cell lines, the regulation of autophagy is frequently altered from that observed in normal cells^43^ which may play a role in the autophagic differences we observed between liver tissue and HepG2 cancer cells. It has previously been suggested that CMA reduction may be compensated for in a tissue-specific manner through upregulation of macroautophagy.^44^ Our data may suggest that, unlike cells in culture that are proliferating, macroautophagy is not adequately upregulated *in vivo* in adult mouse liver to compensate for a reduction in CMA induced by HTT KO, allowing APOE to accumulate. Alternatively, the *in vivo* niche of the liver may also influence its regulation of clearance pathways. It should also be noted that FIP200, a mammalian autophagy protein important for cerebellar health,^45^ is weakly similar to both the C-terminal domain of HTT and to yeast autophagy scaffold Atg11^11^; perhaps with a reduction in HTT function with knockout or polyQ expansion, FIP200 may functionally compensate in a tissue-specific manner.

**Figure 6.**
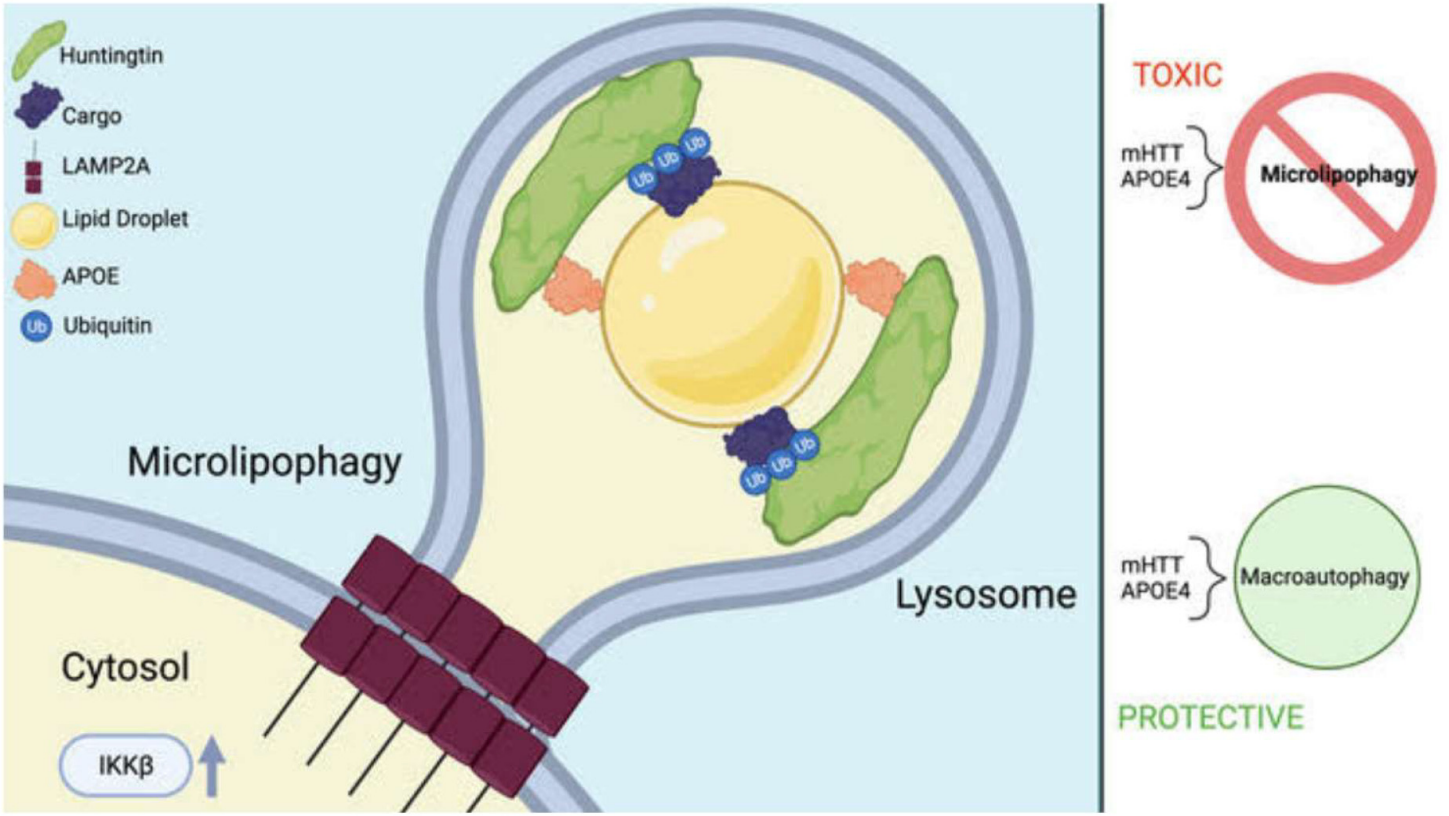
Hypothetical model of a role for HTT in microlipophagy scaffolding ubiquitinated proteins for autophagic degradation. The C-terminal domain of HTT interacts with APOE, which is a lipid droplet surface protein.^33^ The N-terminal domain of HTT interacts with ubiquitinated autophagic cargo,^7^ which become embedded in the associated lipid droplet and shuttled into the lysosome in a LAMP2A-dependent fashion and may overlap with the pathway defined as chaperone-mediated autophagy (CMA). The process may be activated by phosphorylation of HTT serine 13 by IKKbeta.^12,50^ Mutation of HTT or expression of APOE4 may impair this mechanism, which may then be compensated for by upregulation of starvation-induced macroautophagy. Created in BioRender. Lab, T. (2025) https://BioRender.com/r62s317.

HTT-lowering therapies are a promising therapeutic direction that may extend and improve the lives of many HD patients.^46,47^ While the results here are in the context of extensive HTT KD, our work suggests that the tissues targeted for HTT KD should be considered in the design of the clinical trial, as KD of HTT may alter autophagic balance in a tissue-specific way, and may reduce CMA function in human liver as it did here in mice. We recently proposed that HTT may play different roles in microautophagy/CMA, macroautophagy and mitophagy.^7^ APOE is a lipid droplet surface protein that modulates droplet size and triglyceride saturation, the APOE4 variant or APOE KO forming large lipid droplets with impaired turnover contributing to lipotoxicity,^33^ similar to that observed with mutant HTT expression^16^ or a loss of CMA.^48^ We previously proposed a role for the HTT protein in CMA.^7,11^ In Figure 6, we depict a *hypothetical* model in which HTT, APOE and LAMP2A may function together in a form of CMA/microautophagy clearing lipid droplets together with ubiquitinated cargo in the liver, which demonstrates high expression of APOE.^49^

In yeast, microlipophagy employs the autophagic receptor protein Atg39,^41^ which we find has weak sequence similarity to APOE (Supplemental Figure 1). Atg39 interacts directly with the yeast autophagic scaffold protein Atg11, which has similarity to the C-terminal domain of HTT.^6,11,40^ Here, we find that APOE interacts with this domain of HTT, and that APOE accumulates in liver with HTT KO, together with lowered LAMP2A levels reflecting a reduction in CMA, a pathway that clears proteins prone to aggregation.^44^ CMA dysfunction causes toxic protein aggregates to accumulate in the central nervous system and is related to many neurodegenerative diseases such as AD, PD, HD, and ALS.^51^ APOE has recently been shown to aggregate within the endo-lysosomal system of microglia, and to contribute to amyloid beta plaque pathology, a process influenced by microglial lipid metabolism, interferon and the JAK/STAT signaling pathway.^52^ Impairment of lipid homeostasis has been suggested to result in accumulation of protein aggregates.^53^ This may reflect reduced function of CMA/microlipophagy requiring LAMP2A, HTT and APOE resulting in lipid dyshomeostasis. In liver where APOE expression is high, HTT may function as an autophagic scaffold interacting with APOE as a microlipophagy receptor to target ubiquitinated proteins to the lysosome for degradation. This is consistent with our findings showing an ubiquitin-binding domain within HTT that co-purifies with ubiquitinated/ubiquitin-associated RNA-binding proteins^7^ (hypothetical model, Figure 6). This may be similar to the pathway of microlipophagic clearance of ubiquitinated proteins that has been defined in yeast to utilize Atg39.^41^ As yeast Atg39 has also been defined to function as a nucleophagy receptor,^40^ it is possible HTT and APOE may also work together in the autophagic clearance of nuclear proteins by a similar mechanism.

We previously found that APOE is cleared by CMA as well as macroautophagy in liver HepG2 cell culture.^31^ In this study, HTT KO in liver *in vivo* resulted in reduced LAMP2A levels, consistent with an inhibition of CMA. An accumulation of APOE protein in liver may occur with reduced CMA together with inadequate compensatory macroautophagy upregulation. In HepG2, we find HTT KD reduces APOE abundance with activation of macroautophagy, previously shown to occur in a tissue-specific way with failing CMA.^44^ Therefore, in some cell types, a reduction of CMA with HTT KD may result in a compensatory activation of macroautophagy which may be protective, while in other tissues the reduction in CMA may be pathological without adequate activation of compensatory macroautophagy.^7^ This balance may also be altered due to reduced HTT and LAMP2A levels, and reduced autophagy and lysosomal function with aging.^54–57^ The mechanisms of autophagy scaffolded by HTT may be complex and subject to tissue specificity. We suggest here that when designing therapeutic strategies for HD, the autophagic balance of different cell types should be considered.

## Supplemental Material

sj-pdf-1-hun-10.1177_18796397251391110 - Supplemental material for Huntingtin knockdown dysregulates autophagic degradation of Apolipoprotein E Supplemental material, sj-pdf-1-hun-10.1177_18796397251391110 for Huntingtin knockdown dysregulates autophagic degradation of Apolipoprotein E by Gianna M Fote, Nicolette R McClure, Robert M Bragg, Jharrayne McKnight, Leslie M Thompson, Jeffrey B Carroll and Joan S Steffan in Journal of Huntington's Disease
