# Supplementary material for: Huntingtin knockdown dysregulates autophagic degradation of Apolipoprotein E
Source: J Huntingtons Dis. 2025 Oct 29;15(1):110–23. doi: 10.1177/18796397251391110 (PMC12847462; doi:10.1177/18796397251391110)
Supplement: sj-pdf-1-hun-10.1177_18796397251391110 - Supplemental material for Huntingtin knockdown dysregulates autophagic degradation of Apolipoprotein E [file sj-pdf-1-hun-10.1177_18796397251391110.pdf]

Supplemental Material

Huntingtin knockdown dysregulates autophagic degradation of Apolipoprotein E

Supplemental Figure 1. Potential conservation of APOE across phylogeny to function as autophagy receptor Atg39. The black arrows are adjacent to human APOE. Alignments were done using EMBL-EBI MUltiple Sequence Comparison by Log-Expectation (MUSCLE) [1,2] and Jalview Java Alignment Editor. [3,4] The "Conservation" bars show amino acid property conservation, the "Quality" bars denote BLOSUM62 score based on observed substitutions, and the "Consensus" shows the commonest residues and their percentage for each column of the multialignment.

References

1. Edgar RC. MUSCLE: a multiple sequence alignment method with reduced time and space complexity. *BMC Bioinformatics* 2004; 5: 113. 20040819. DOI: 10.1186/1471-2105-5-113.
2. Edgar RC. MUSCLE: multiple sequence alignment with high accuracy and high throughput. *Nucleic Acids Res* 2004; 32: 1792-1797. 20040319. DOI: 10.1093/nar/gkh340.
3. Clamp M, Cuff J, Searle SM, et al. The Jalview Java alignment editor. *Bioinformatics* 2004; 20: 426-427. 20040122. DOI: 10.1093/bioinformatics/btg430.
4. Waterhouse AM, Procter JB, Martin DM, et al. Jalview Version 2--a multiple sequence alignment editor and analysis workbench. *Bioinformatics* 2009; 25: 1189-1191. 20090116. DOI: 10.1093/bioinformatics/btp033.

Potential conservation of Apolipoprotein E across phylogeny to function as autophagy receptor Atg39

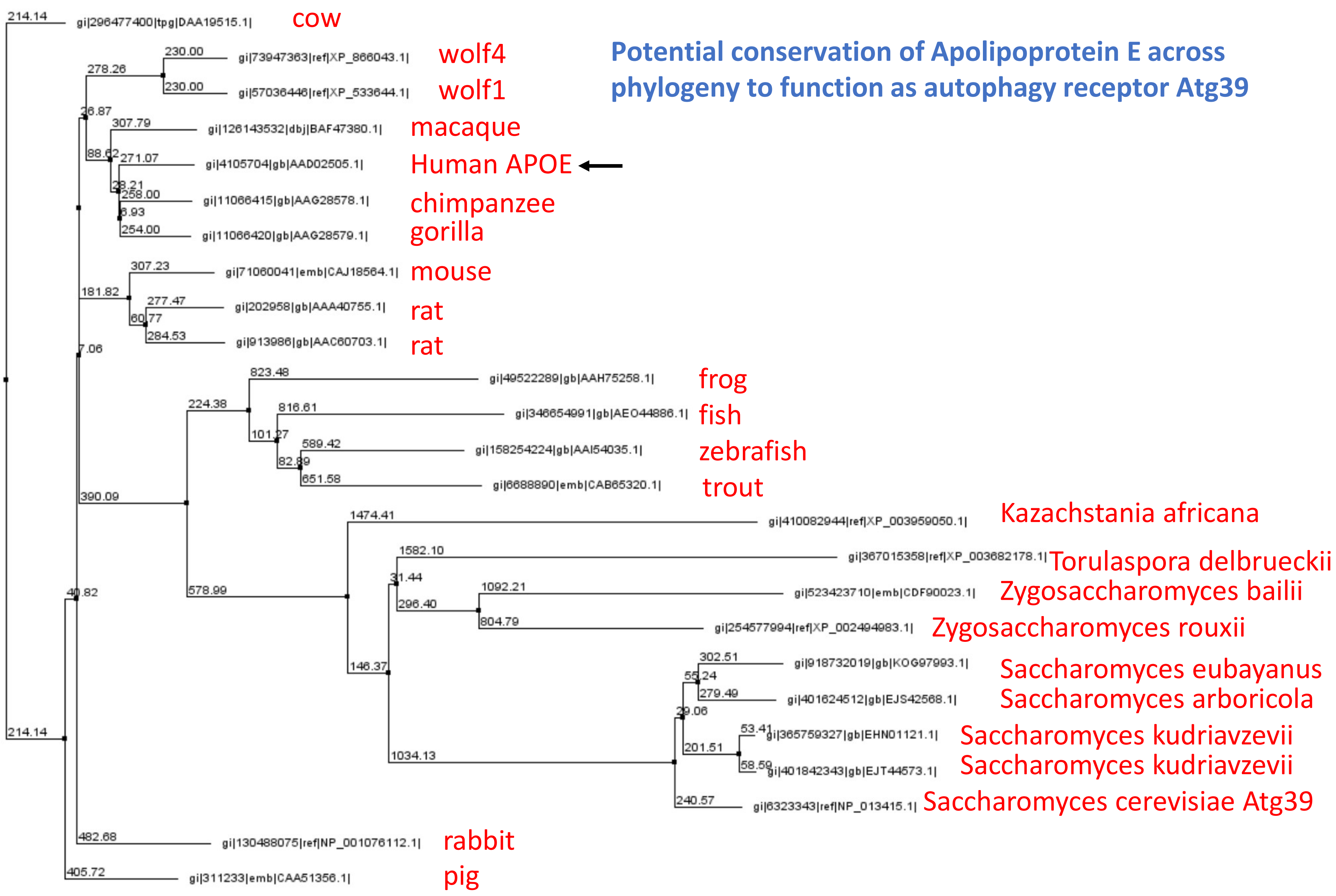

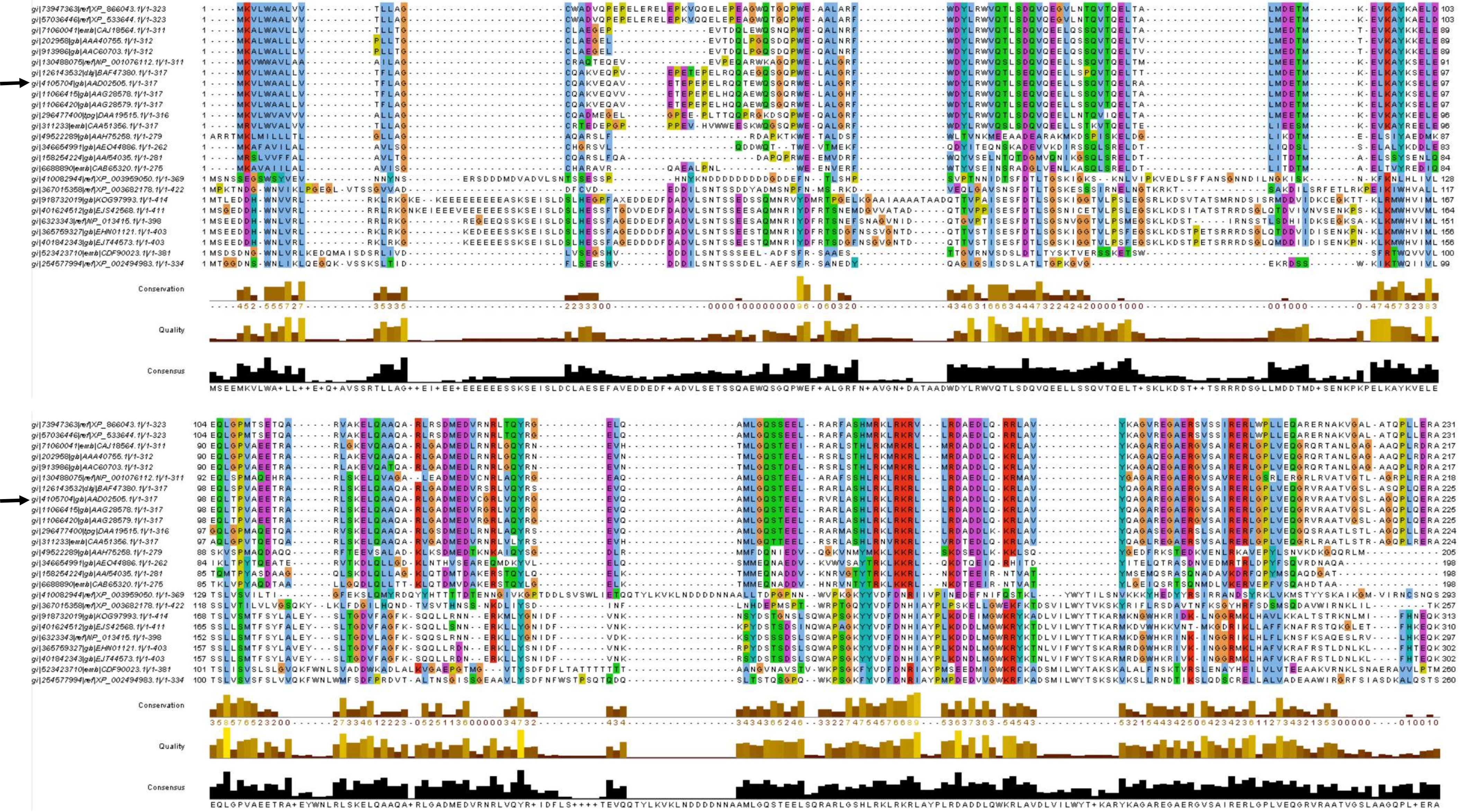
Conservation
Quality
Consensus

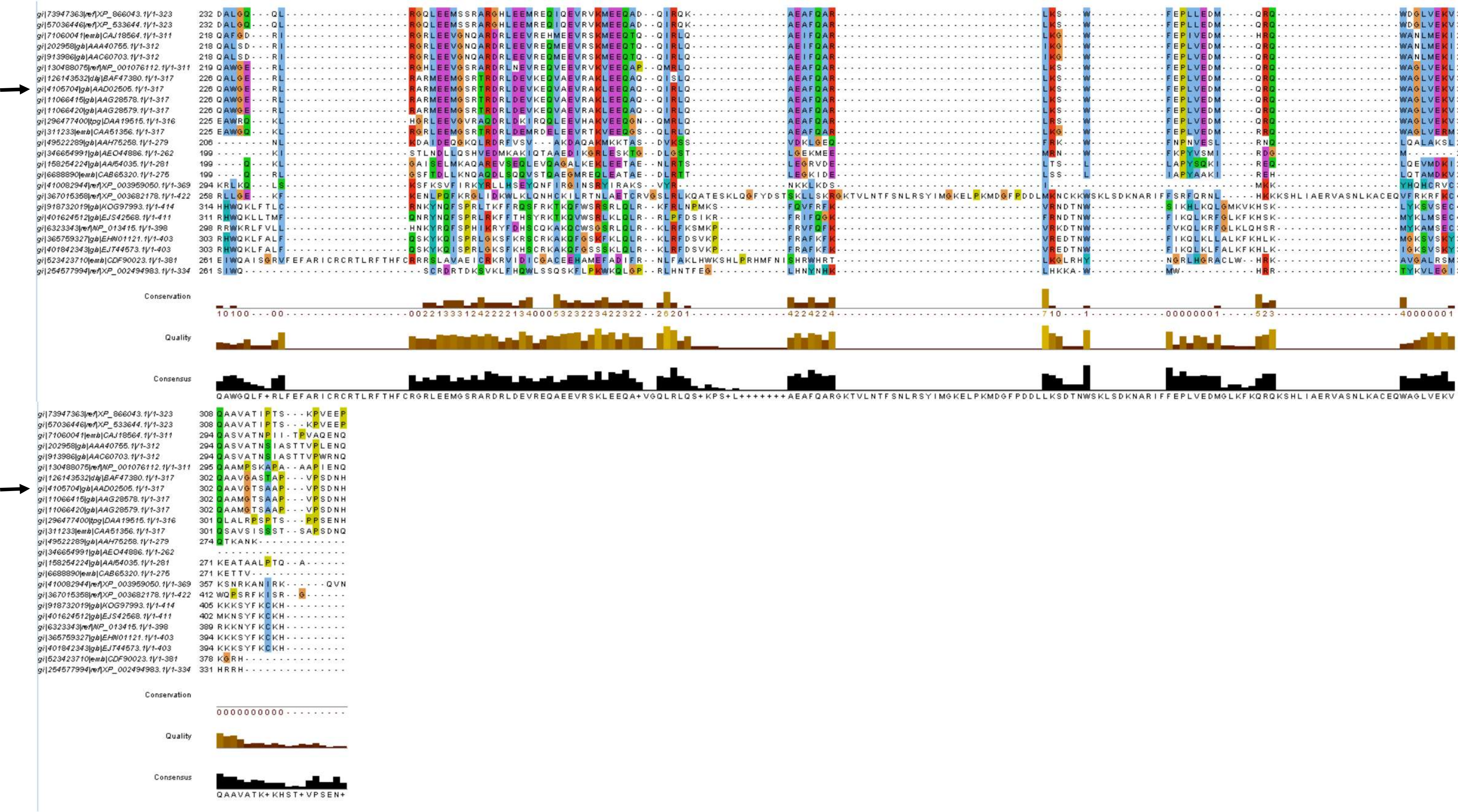
Conservation
Quality
Consensus
Conservation
Quality
Consensus

Supplemental Material

Huntingtin knockdown dysregulates autophagic degradation of Apolipoprotein E

Supplemental Figure 2. Uncropped western blot gel images for Figures 1-5.

Supplementary Figure 2

Uncropped blots for Figure 1A

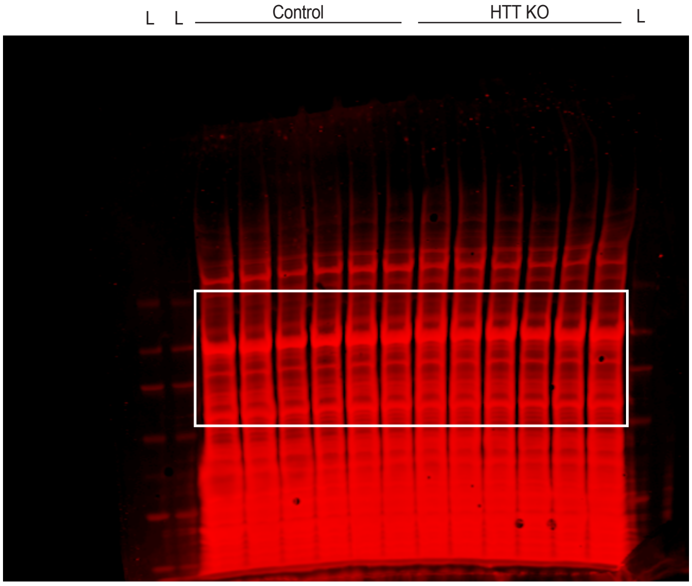

Female: REVERT total protein stain

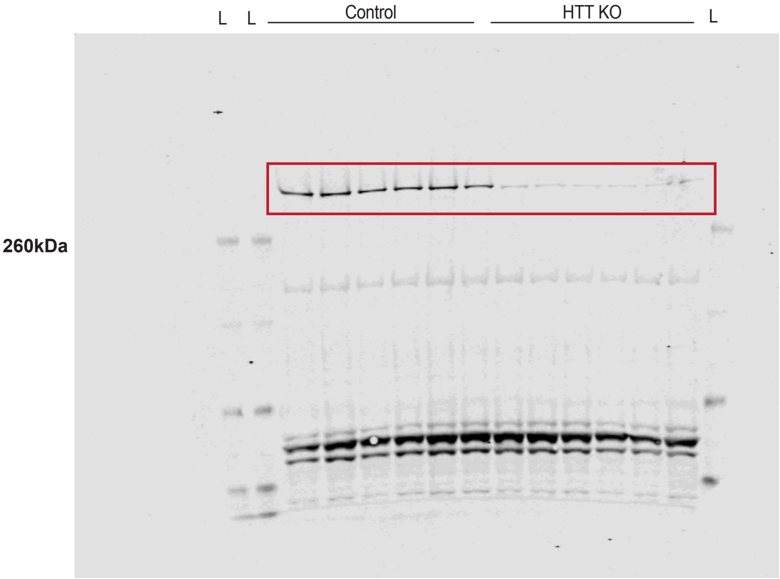

Female: IB: HTT (bw)

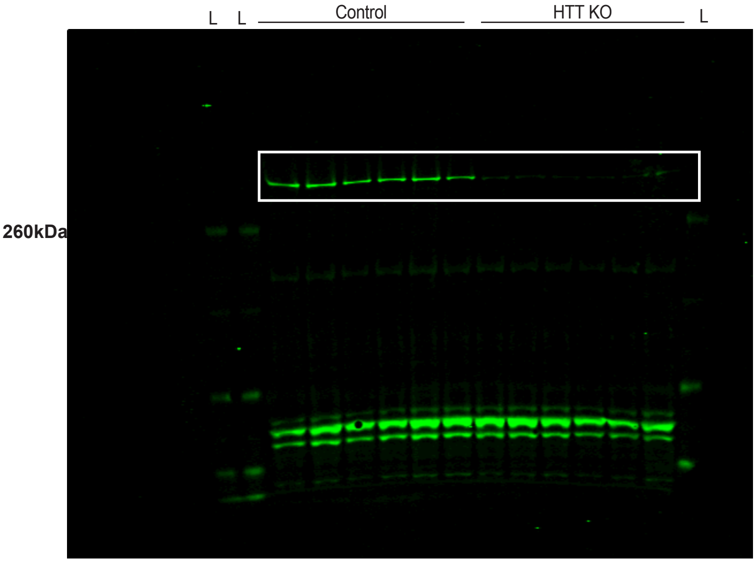

Female: IB: HTT (green)

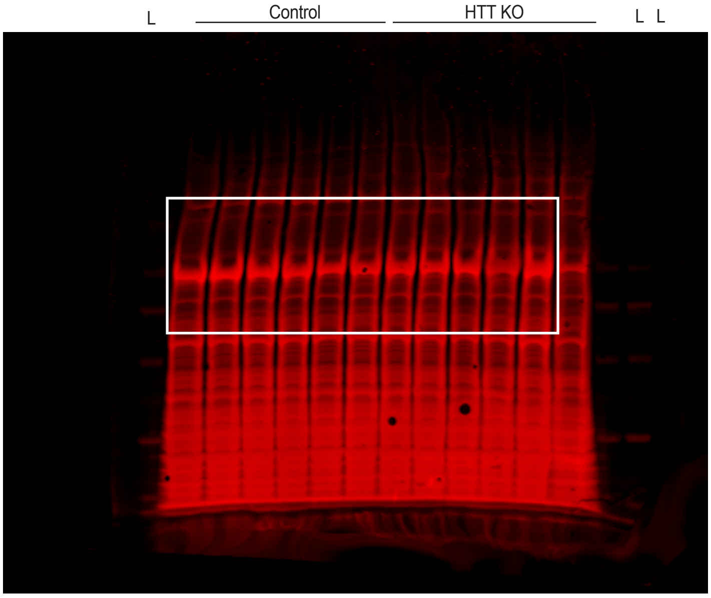

Male: REVERT total protein stain

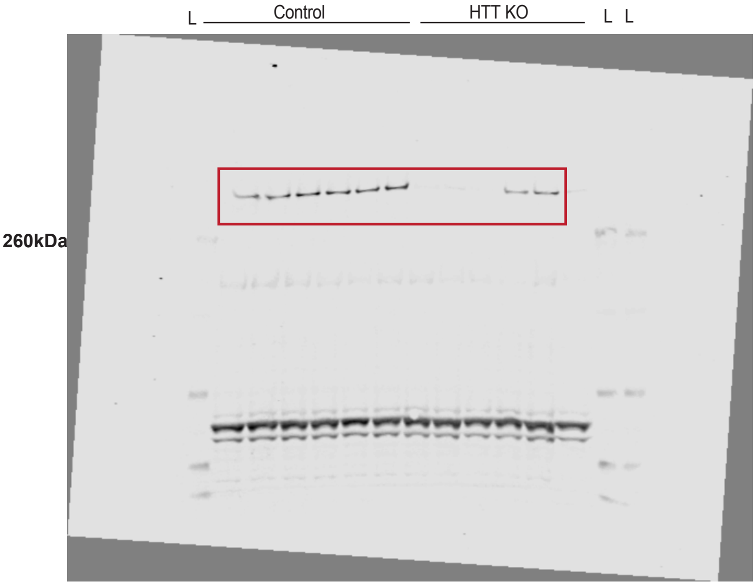

Male: IB: HTT (bw)

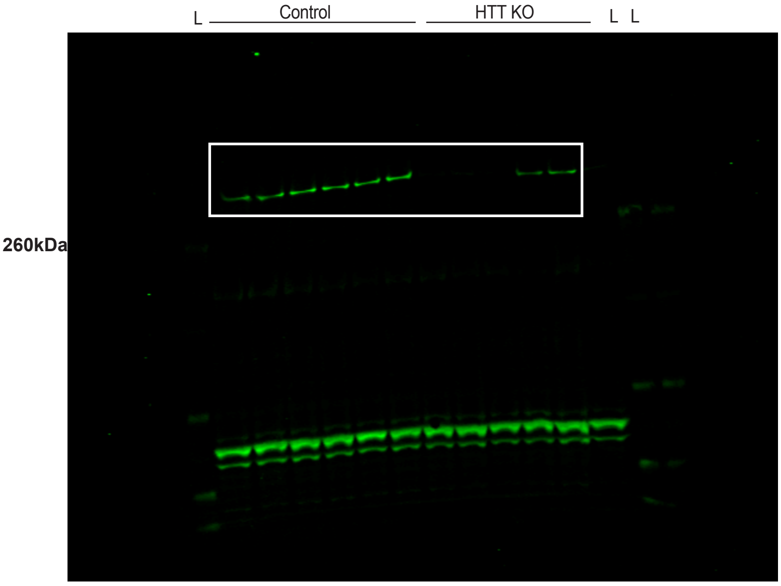

Male: IB: HTT (green)

Uncropped blots for Figure 1B

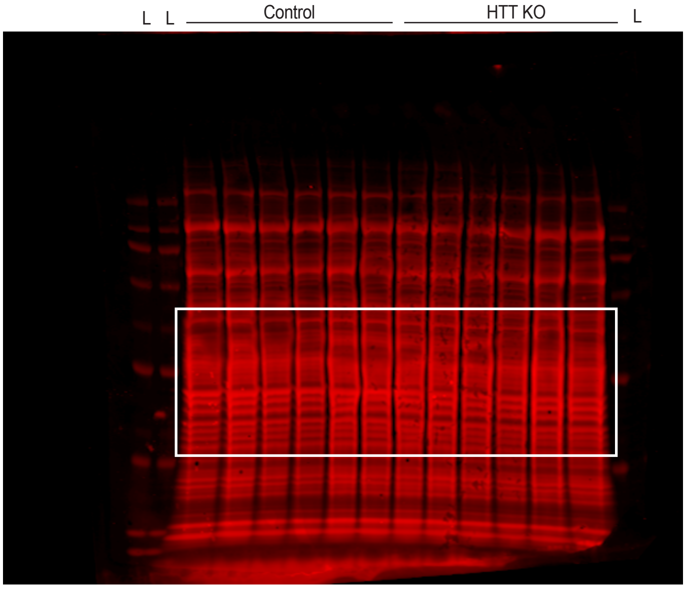

Female: REVERT total protein stain

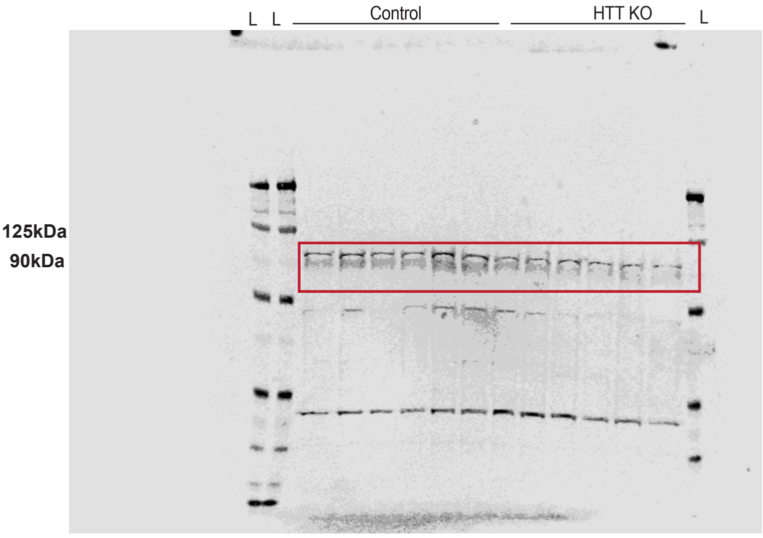

Female: IB: Lamp2A (bw)

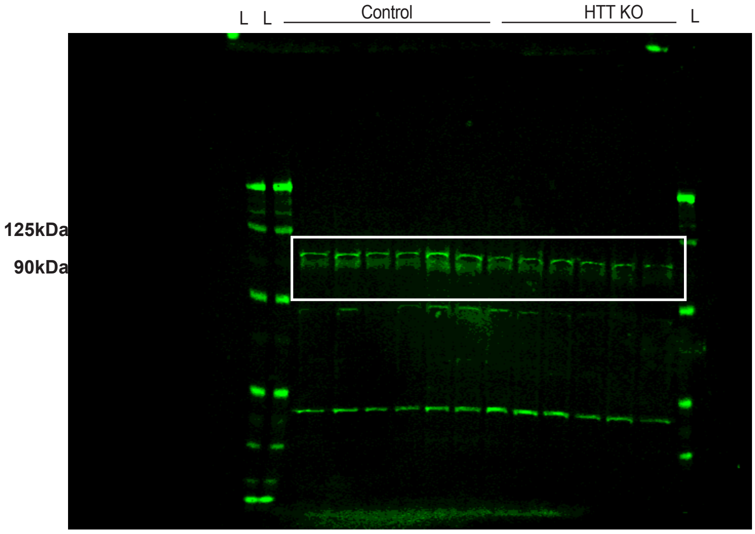

Female: IB: Lamp2A(green)

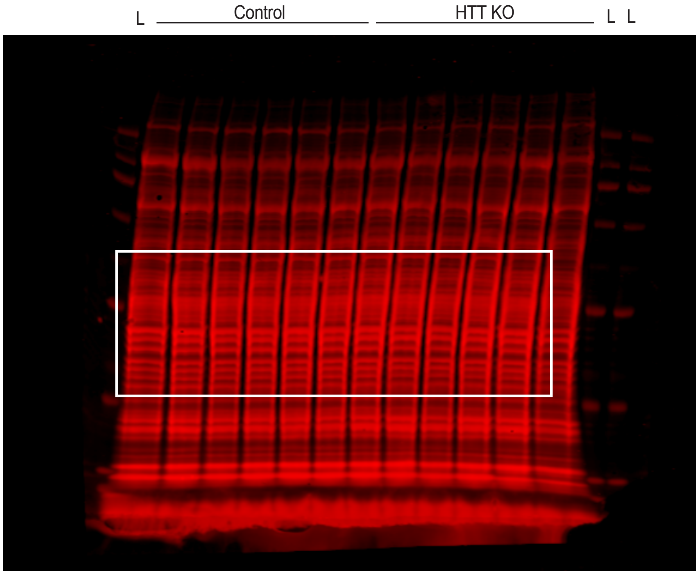

Male: REVERT total protein stain

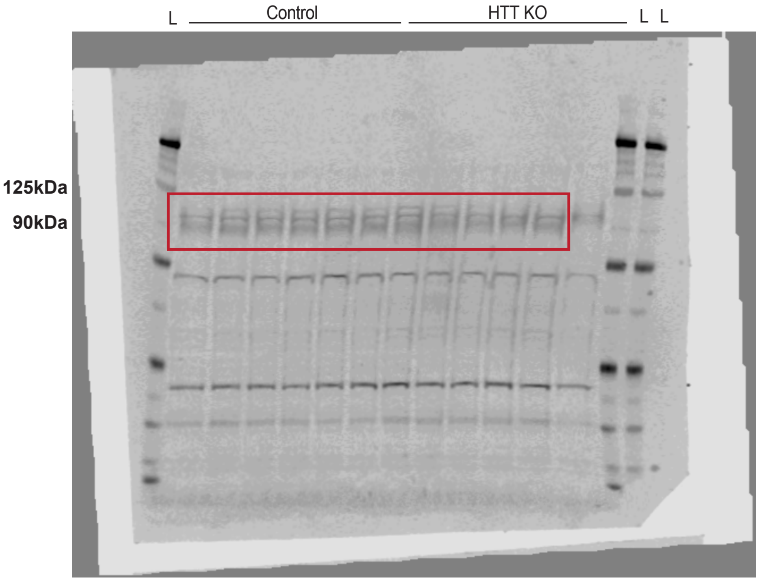

Male: IB: Lamp2A(bw)

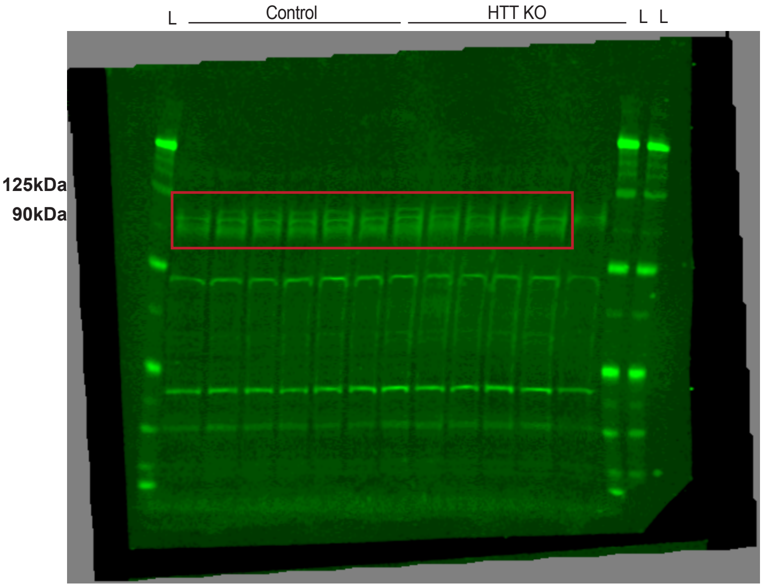

Male: IB: Lamp2A(green)

Uncropped blots for Figure 1C

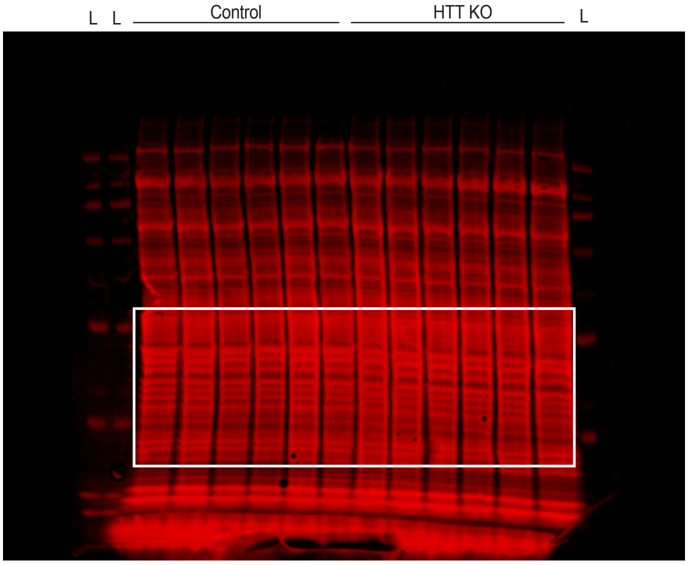

Female: REVERT total protein stain

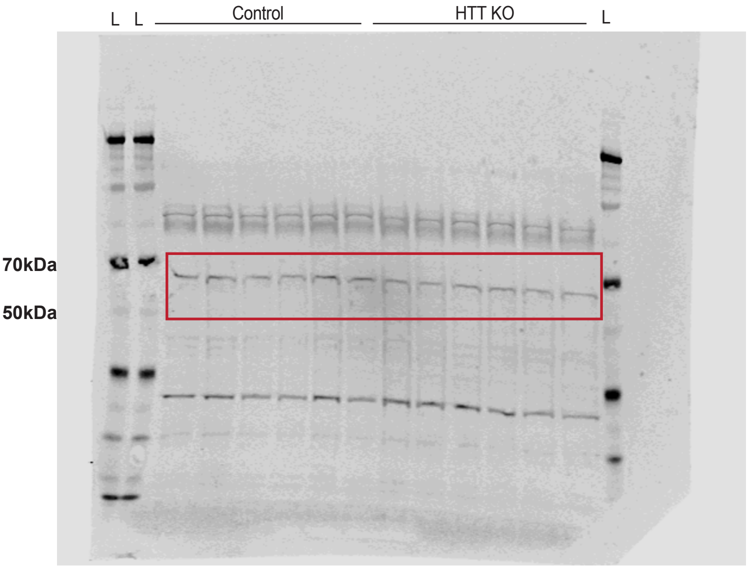

Female: IB: p62(bw)

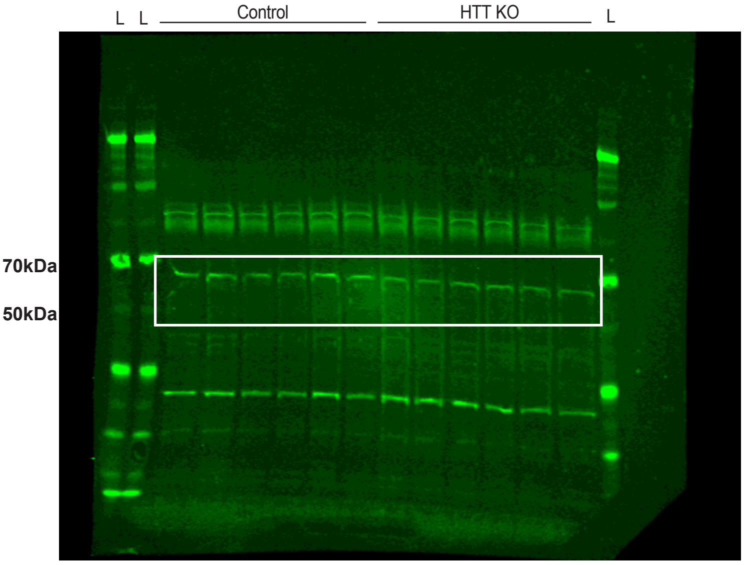

Female: IB: p62(green)

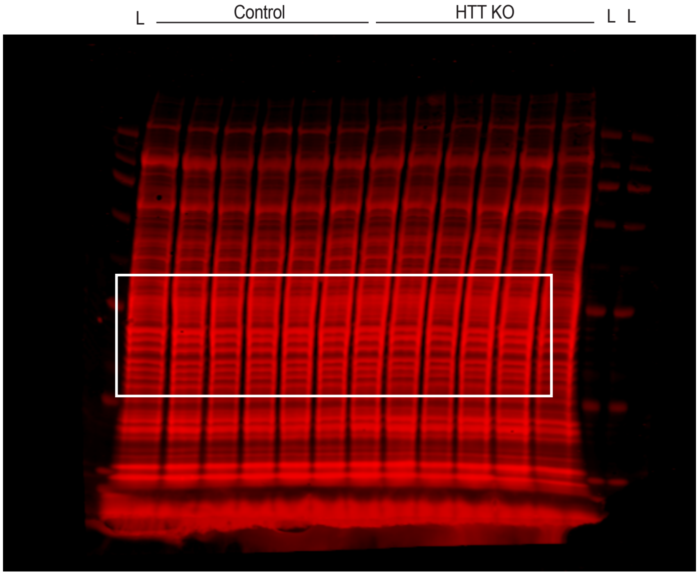

Male: REVERT total protein stain

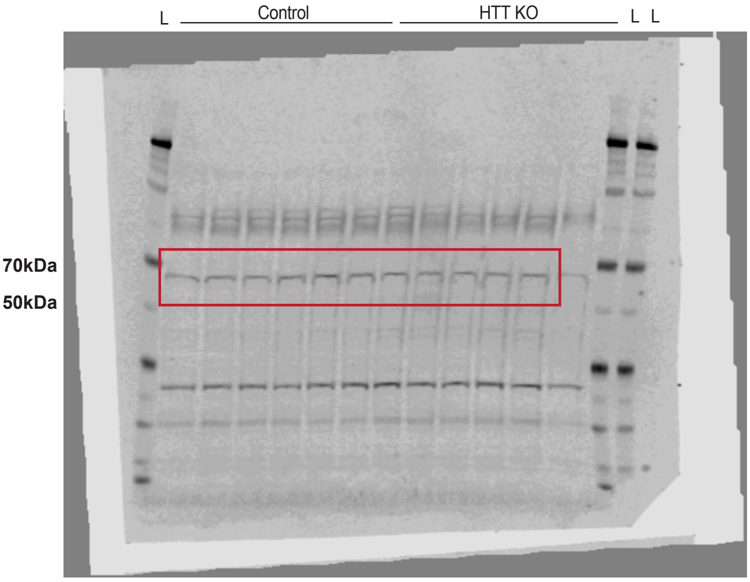

Male: IB: p62(bw)

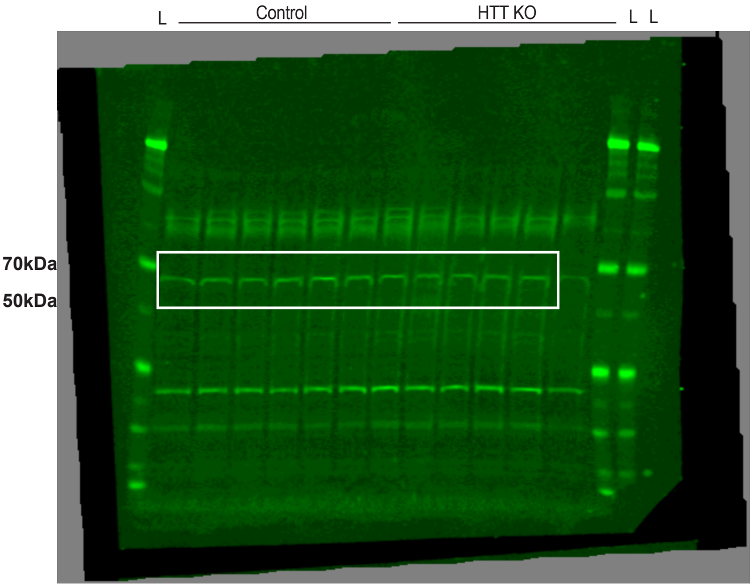

Male: IB: p62(green)

Uncropped blots for Figure 1D

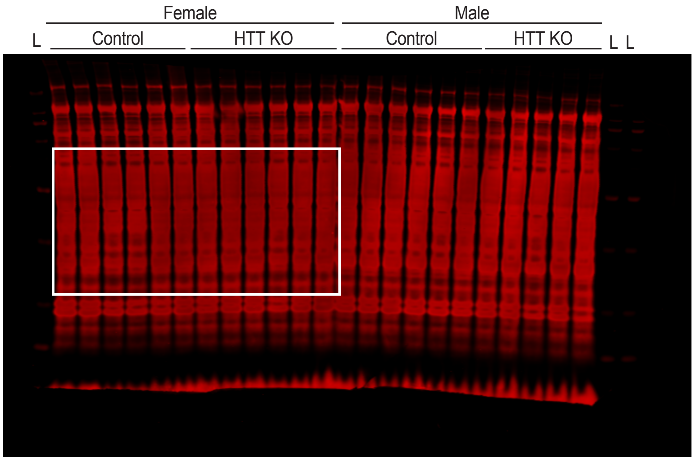

Female: REVERT total protein stain

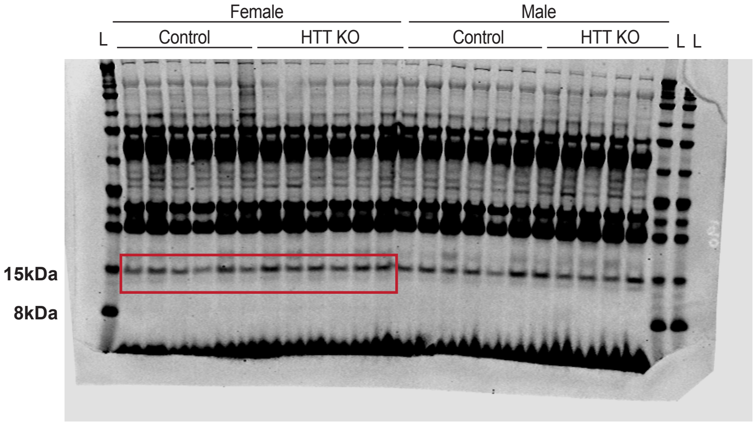

Female: IB: LC3(bw)

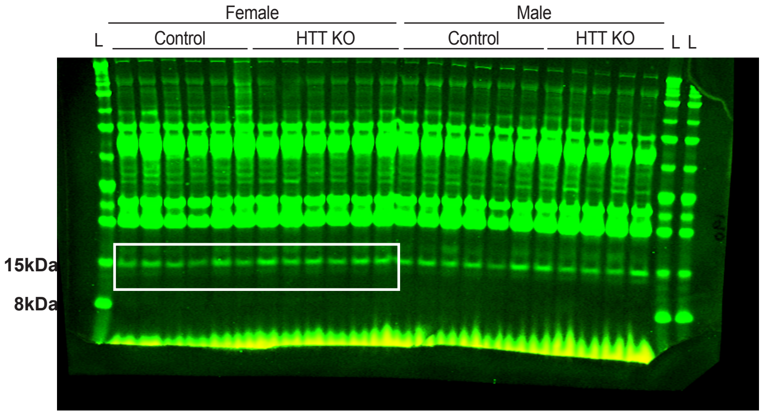

Female: IB: LC3(green)

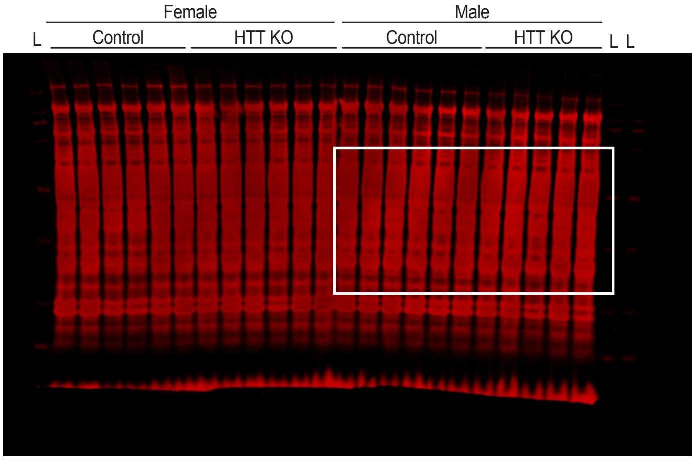

Male: REVERT total protein stain

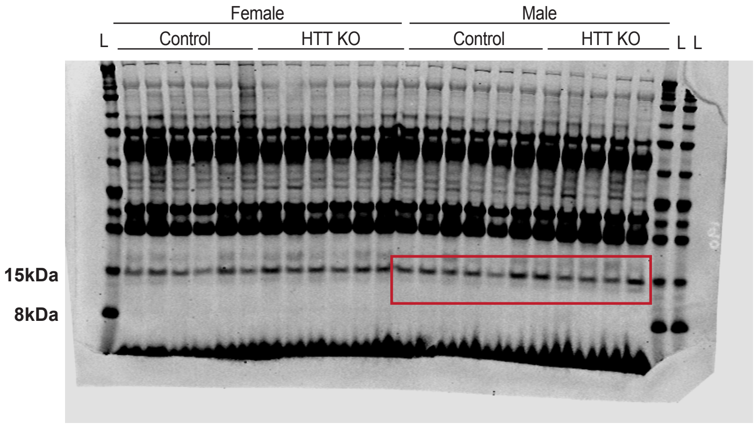

Male: IB: LC3(bw)

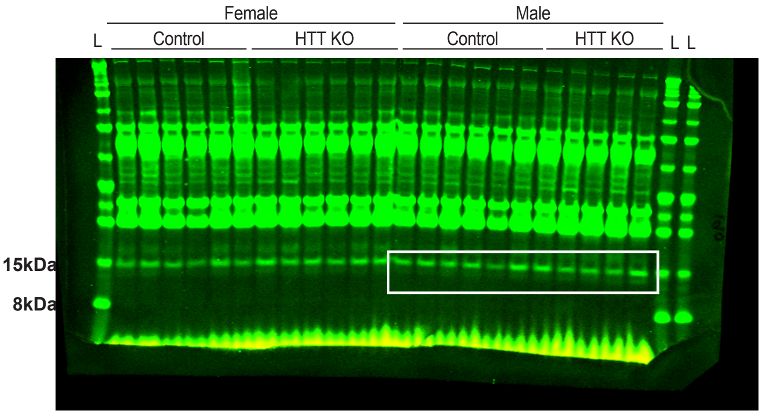

Male: IB: LC3(green)

Uncropped blots for Figure 1E

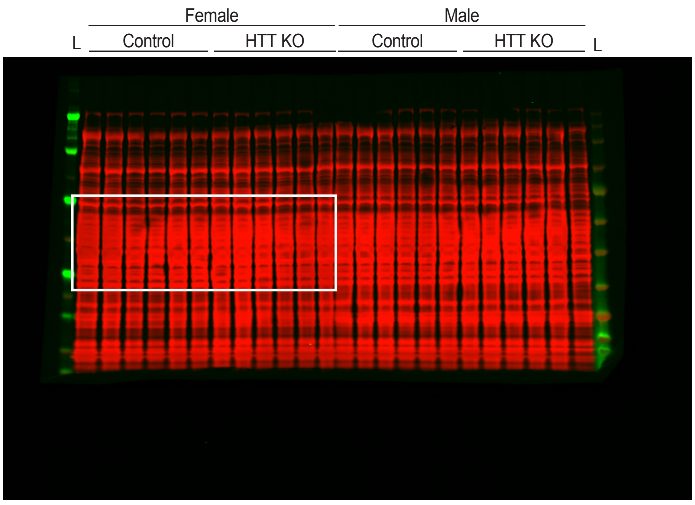

Female: REVERT total protein stain

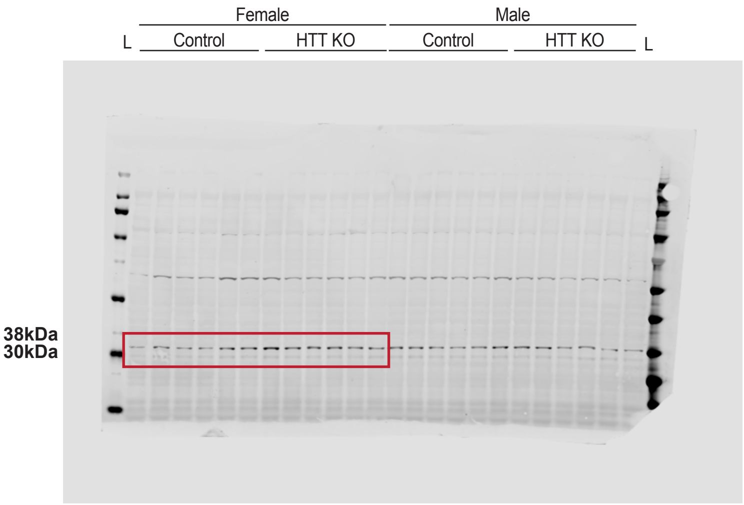

Female: IB: APOE (bw)

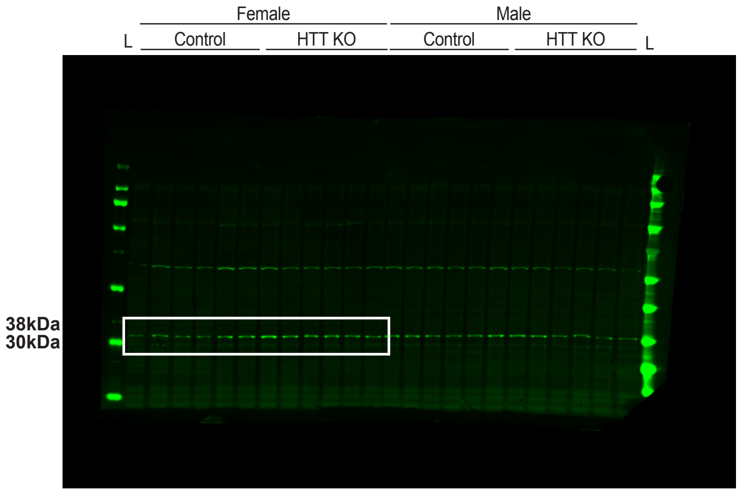

Female: IB: APOE (green)

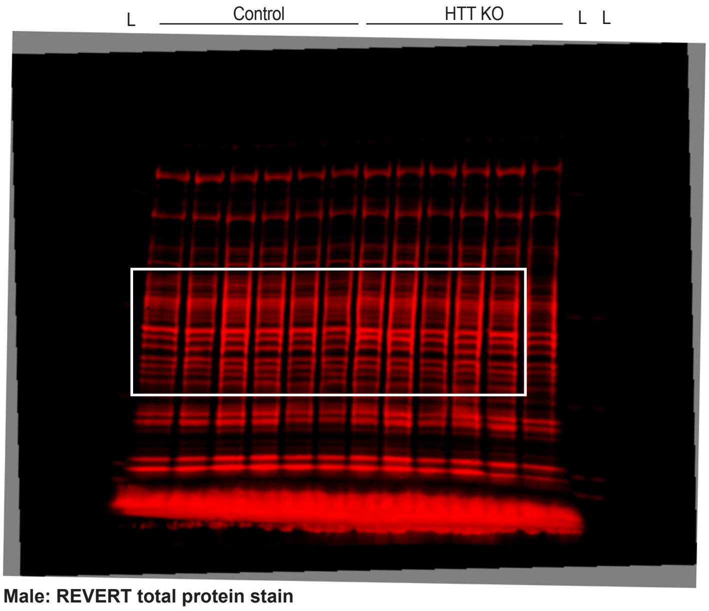

Male: REVERT total protein stain

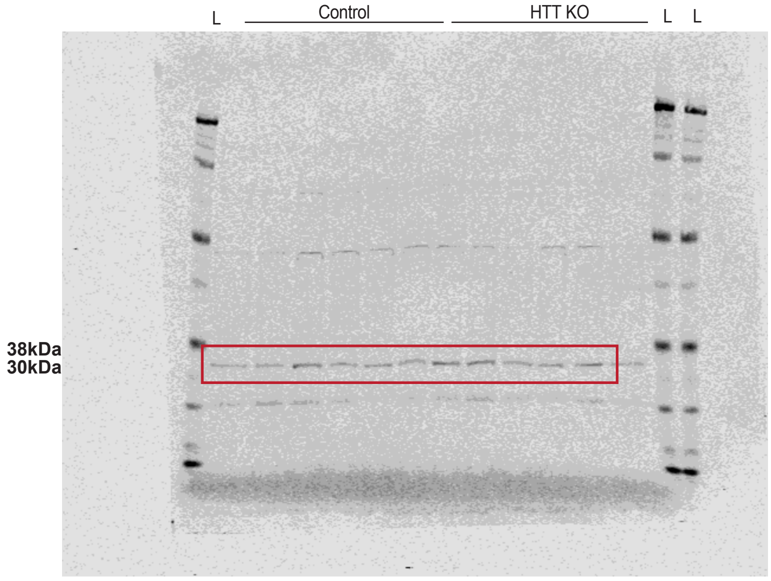

Male: IB: APOE (bw)

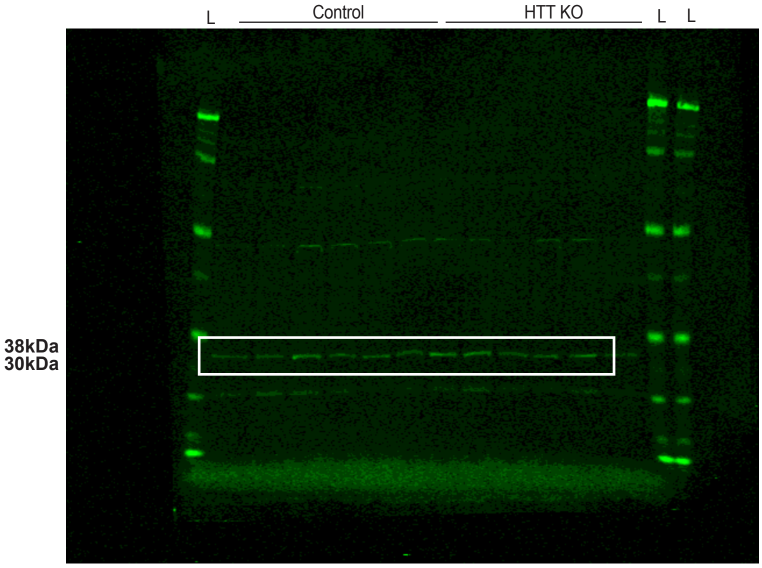

Male: IB: APOE (green)

Uncropped blots for Figure 2A

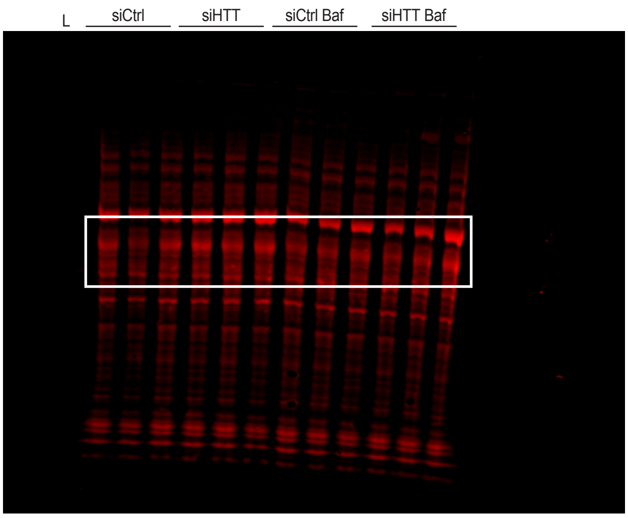

REVERT total protein stain

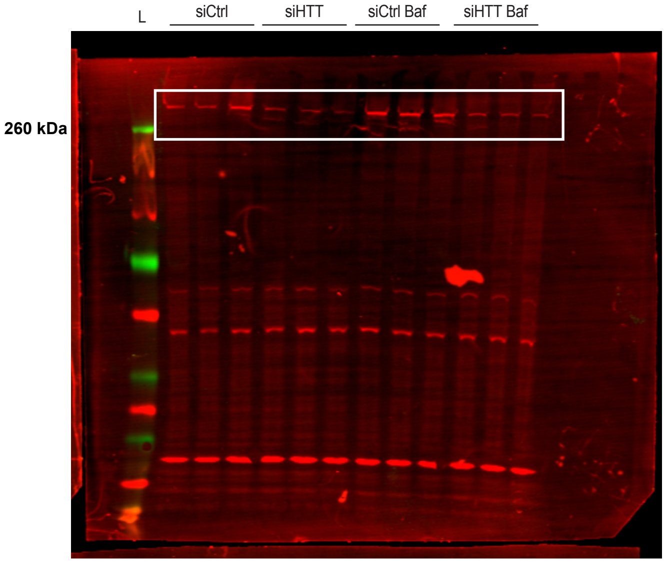

IB: HTT (red)

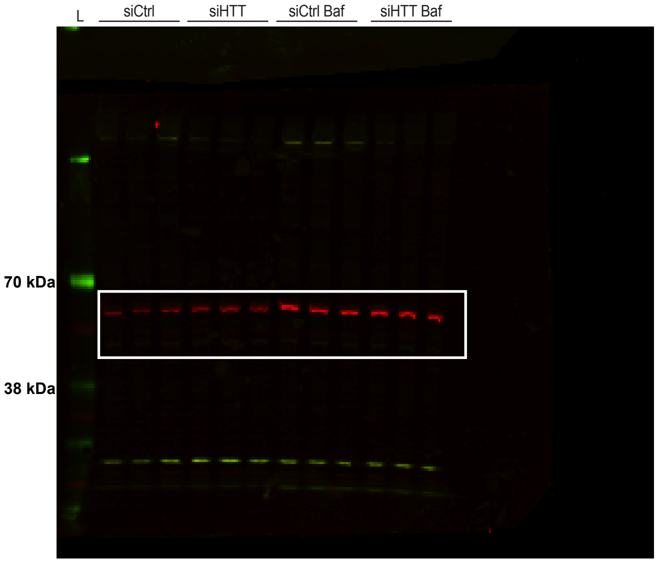

IB: p62 (red)

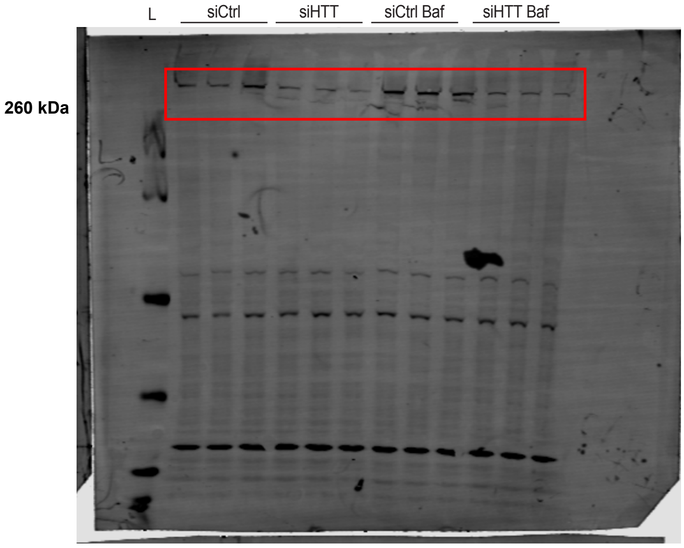

IB: HTT (bw)

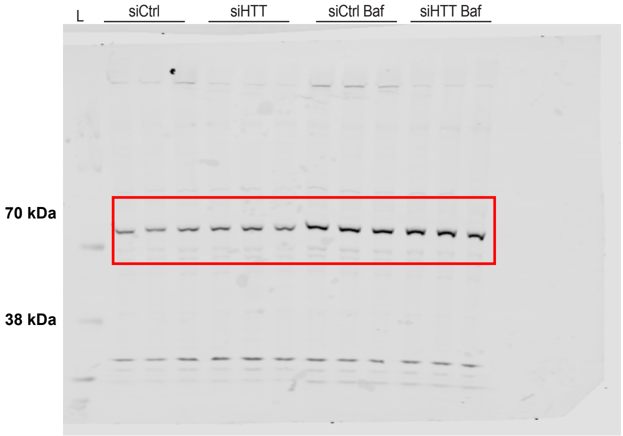

IB: p62 (bw)

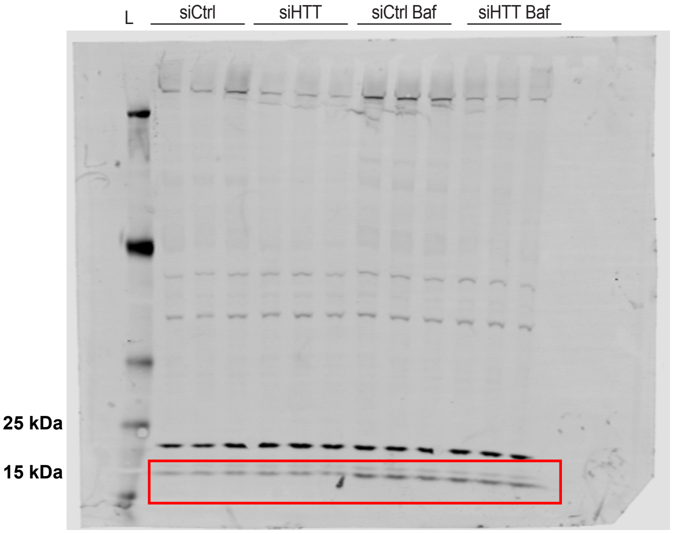

IB: LC3 (bw)

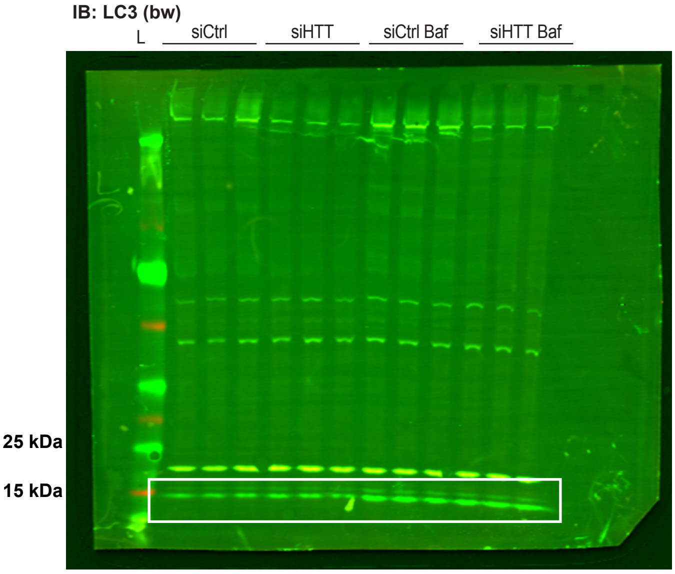

IB: LC3 (green)

Uncropped blots for Figure 2B

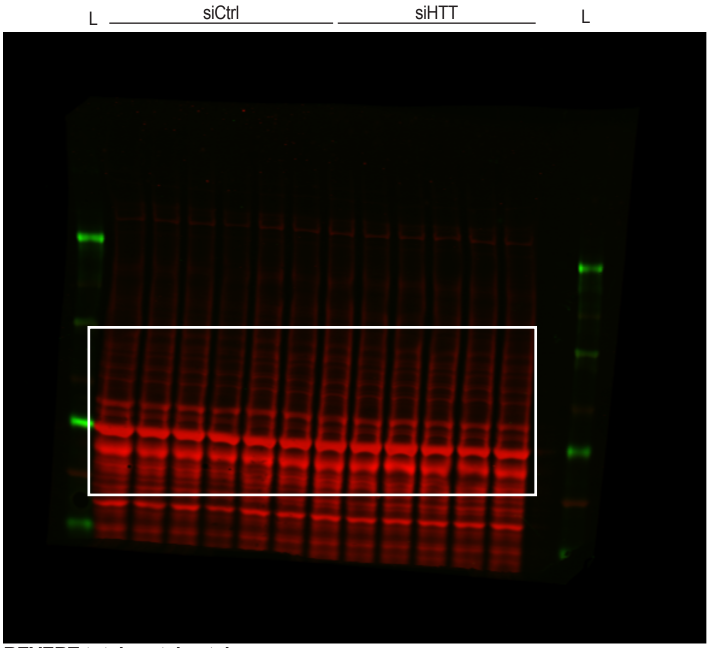

REVERT total protein stain

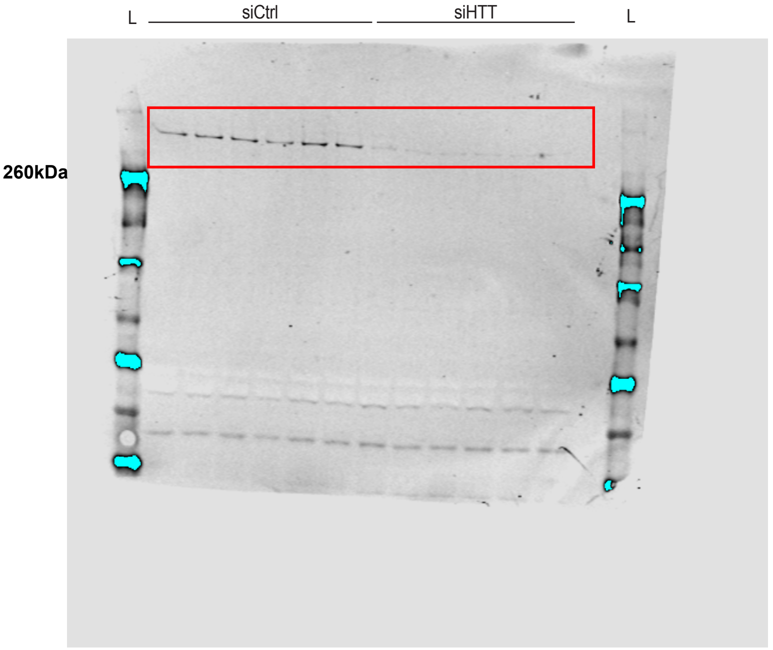

IB: HTT (bw)

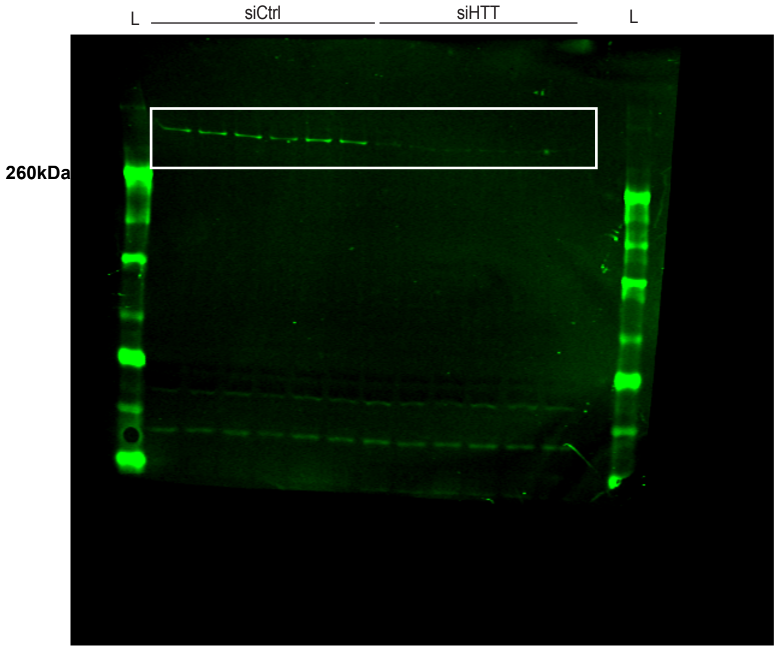

IB: HTT (green)

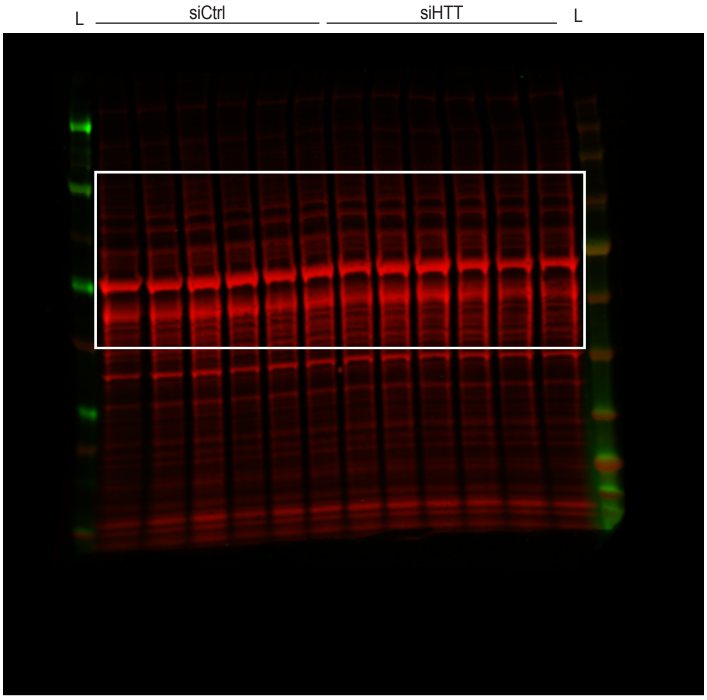

REVERT total protein stain

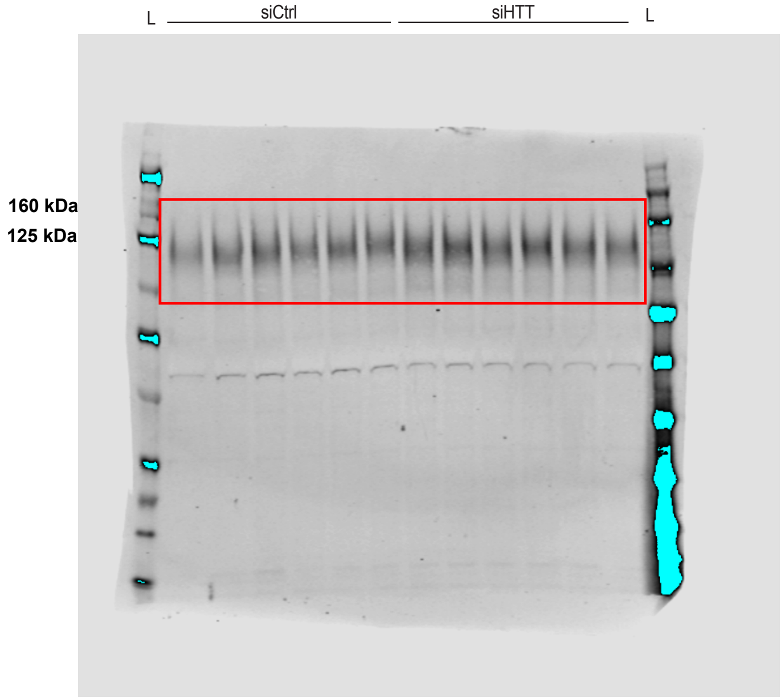

IB: Lamp2A (bw)

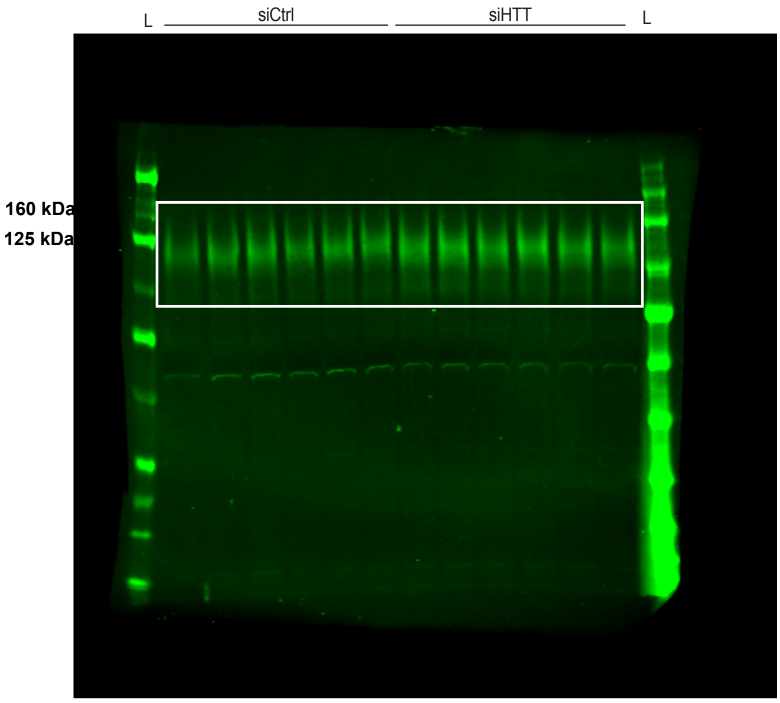

IB: Lamp2A (green)

Uncropped blots for Figure 3A

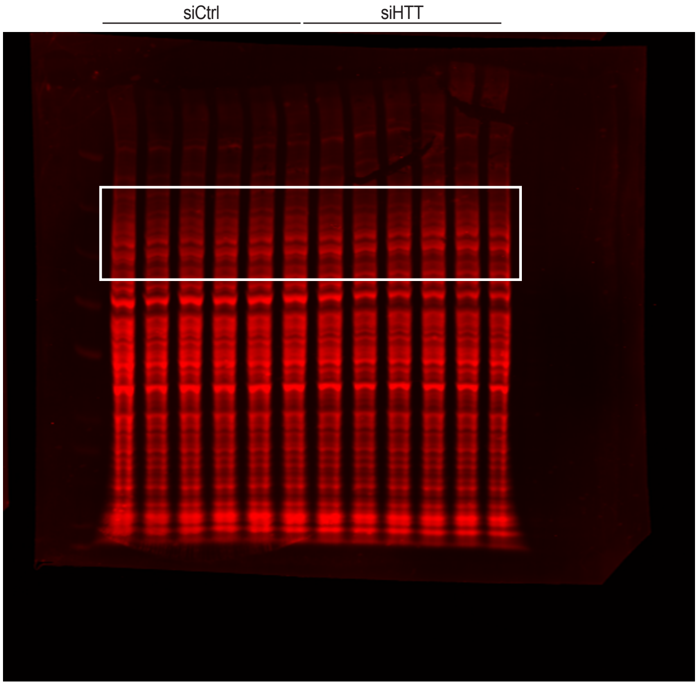

REVERT total protein stain

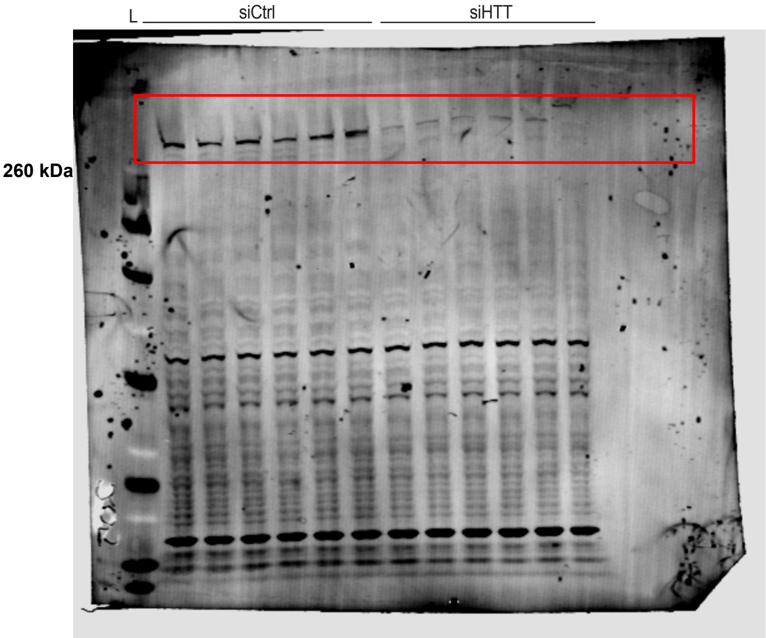

IB: HTT (bw)

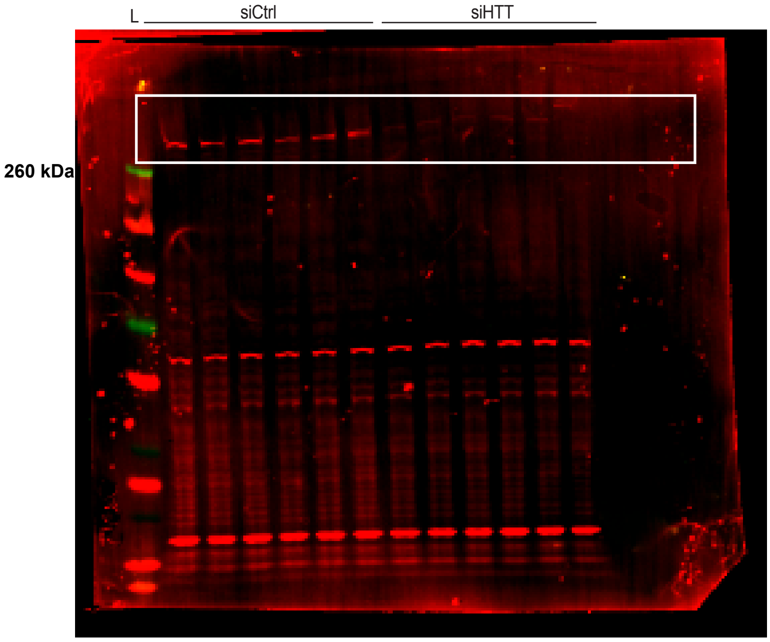

IB: HTT (red)

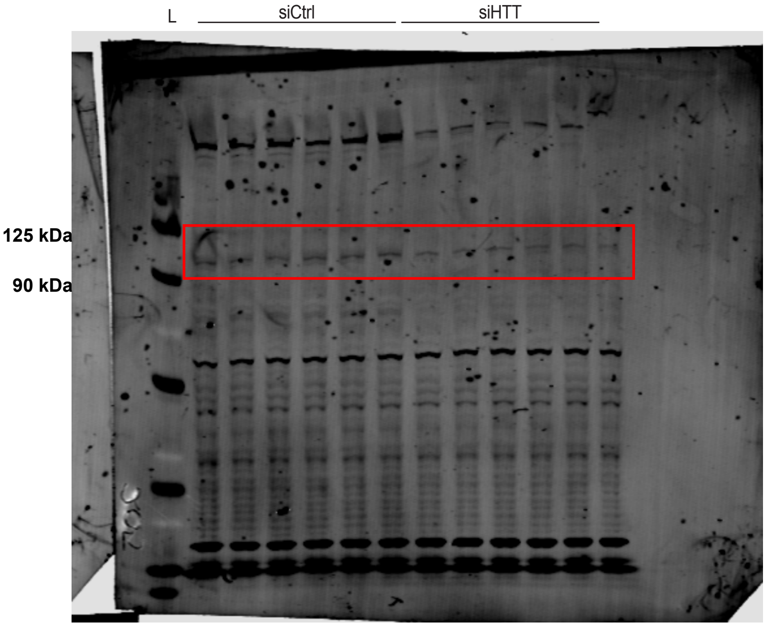

IB: LDLR

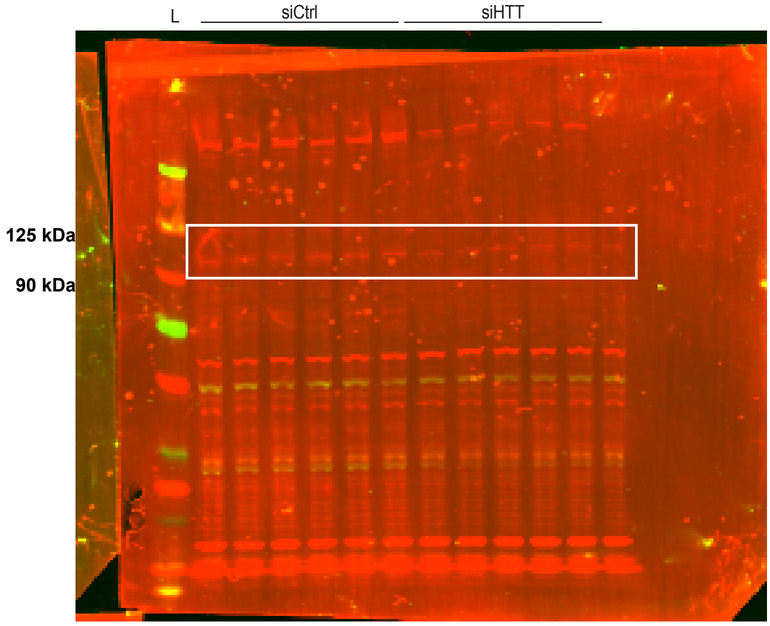

IB: LDLR

Uncropped blots for Figure 3A

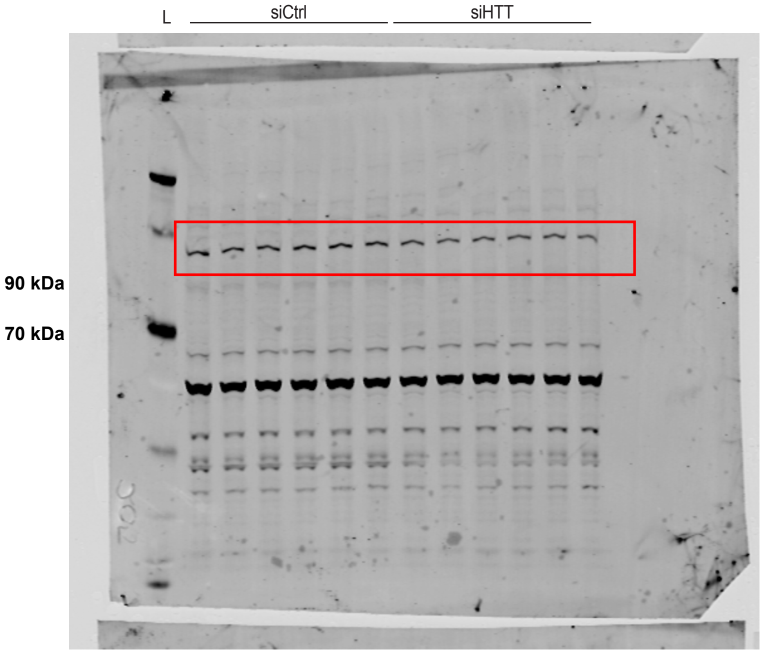

IB: LRP1 (bw)

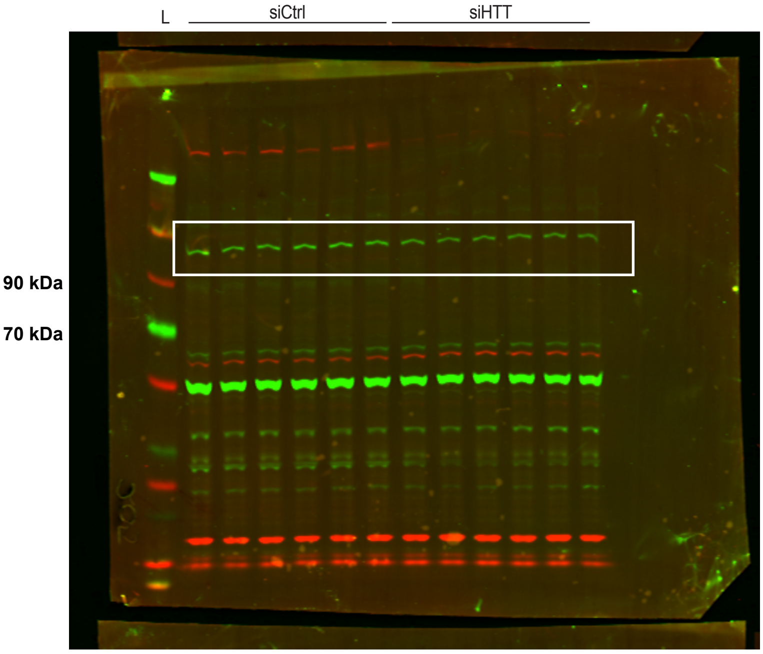

IB: LRP1 (green)

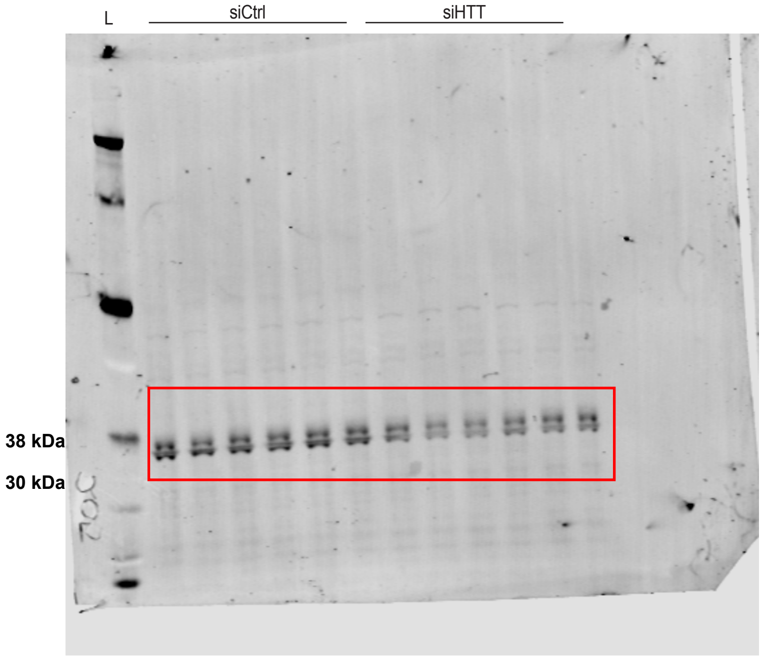

IB: APOE (bw)

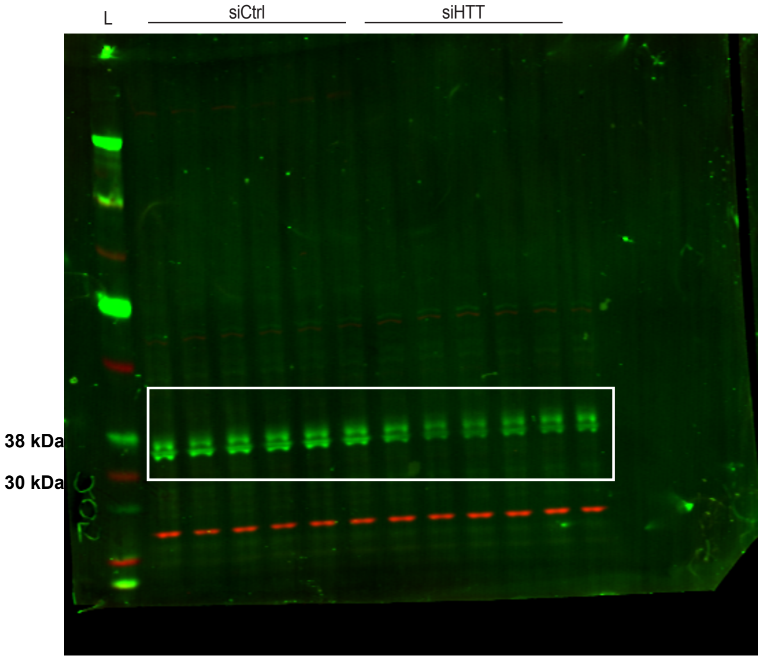

IB: APOE (green)

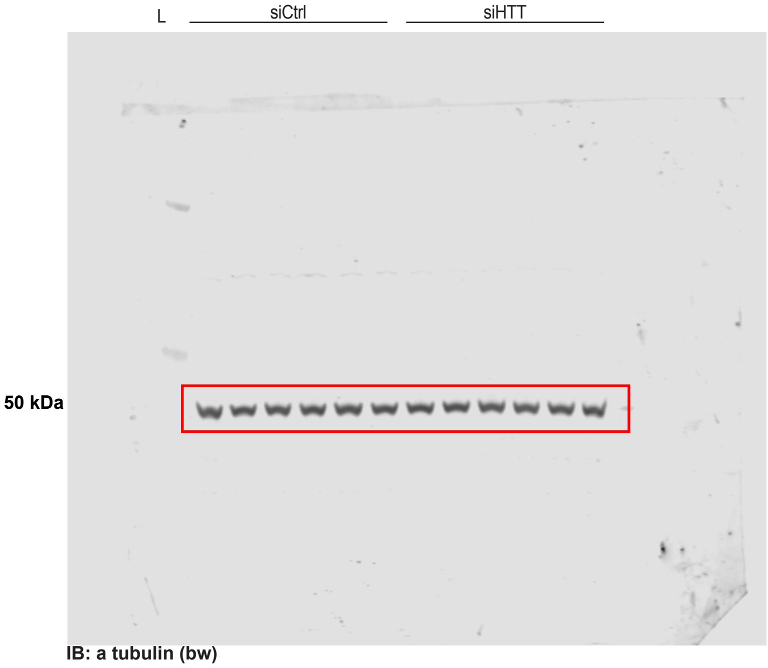

IB: a tubulin (bw)

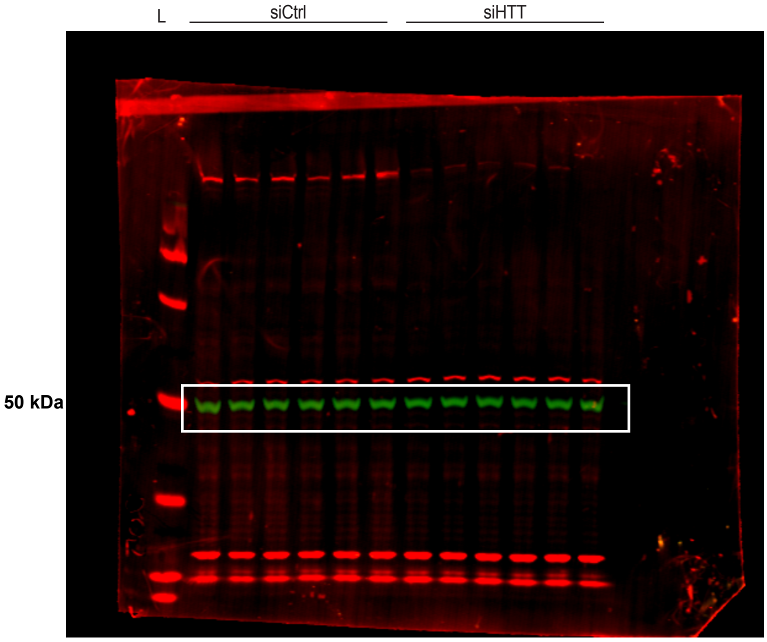

IB: a tubulin (green)

Uncropped blots for Figure 3B

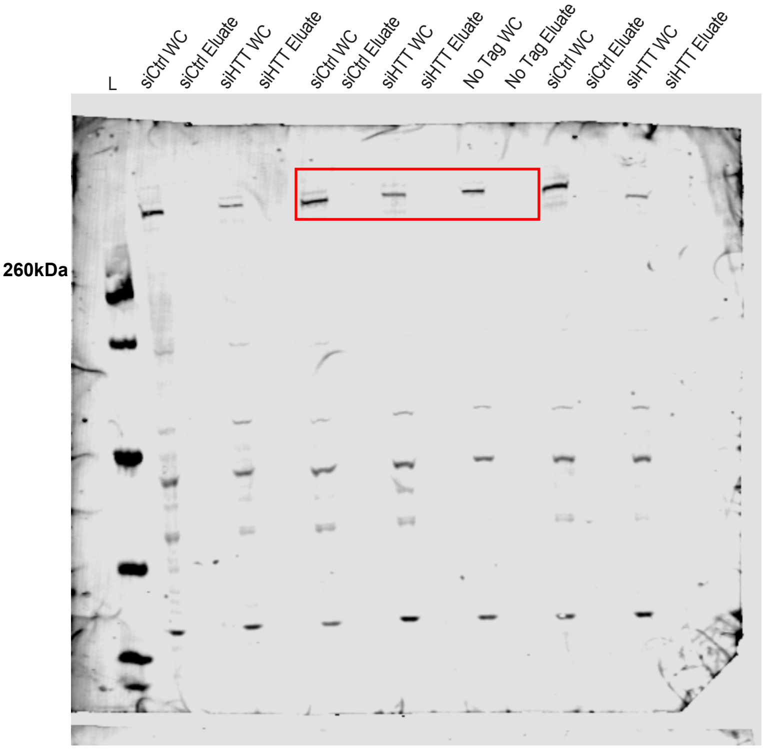

IB: HTT (bw)

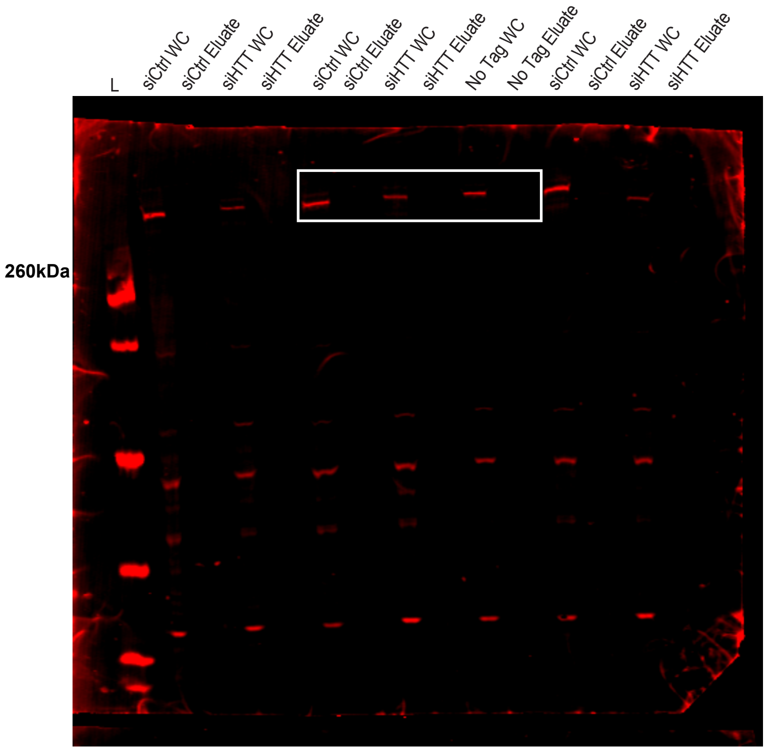

IB: HTT (red)

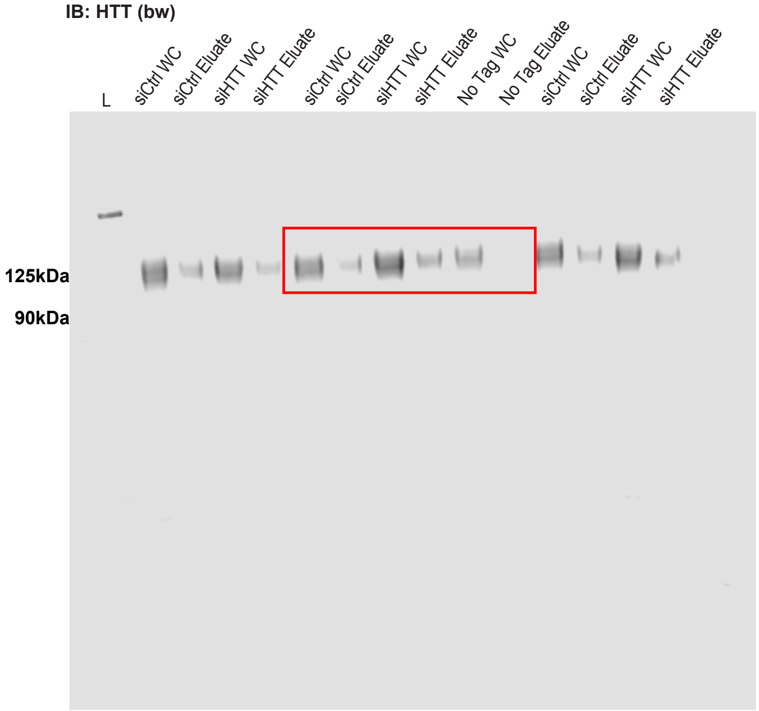

IB: LAMP1 (bw)

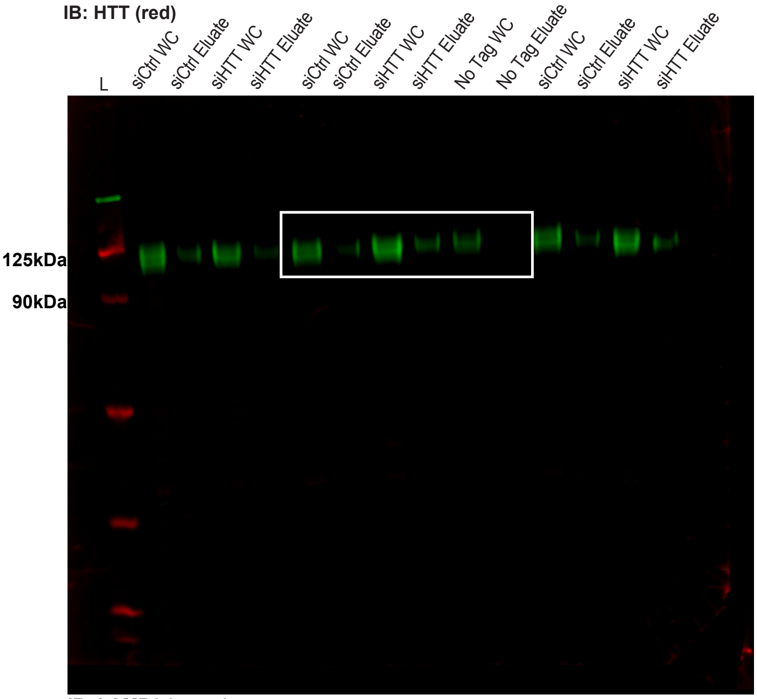

IB: LAMP1 (green)

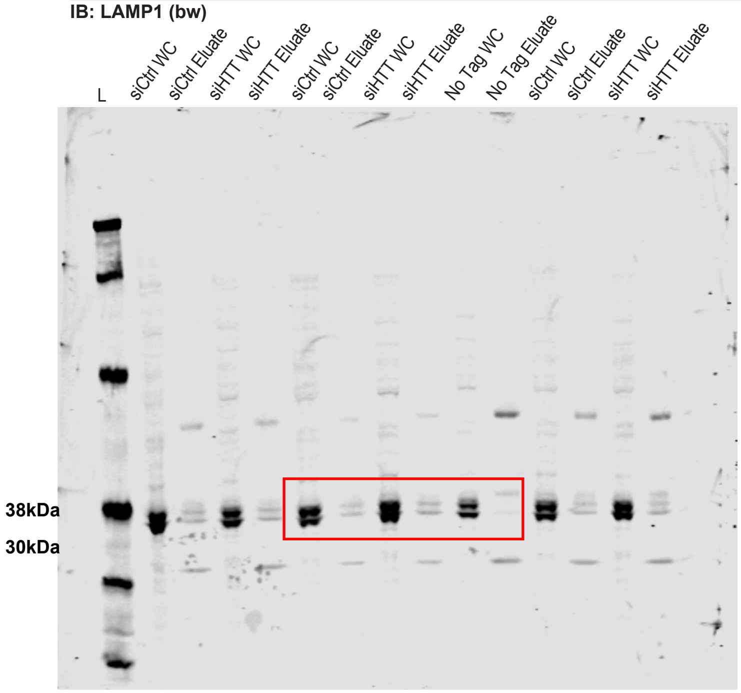

IB: APOE (bw)

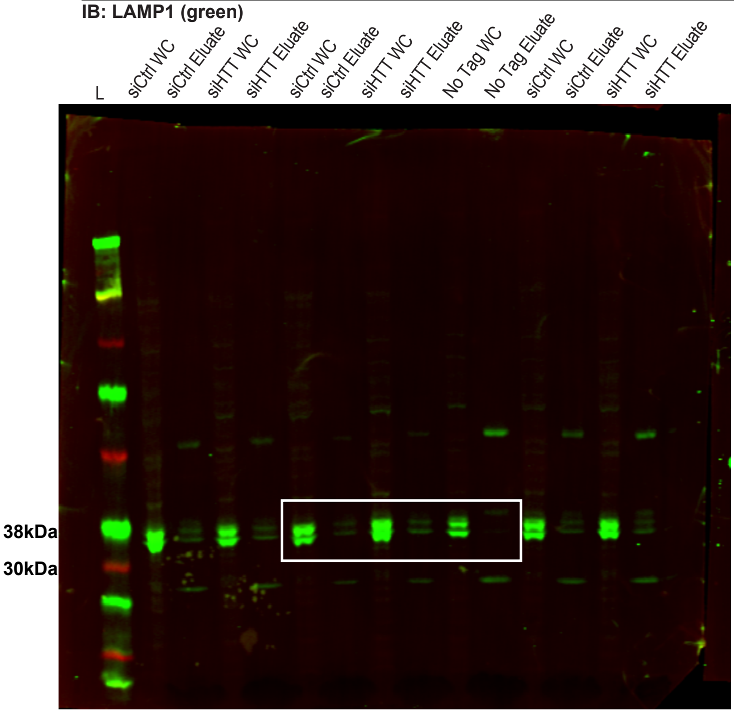

IB: APOE (green)

Uncropped blots for Figure 3C

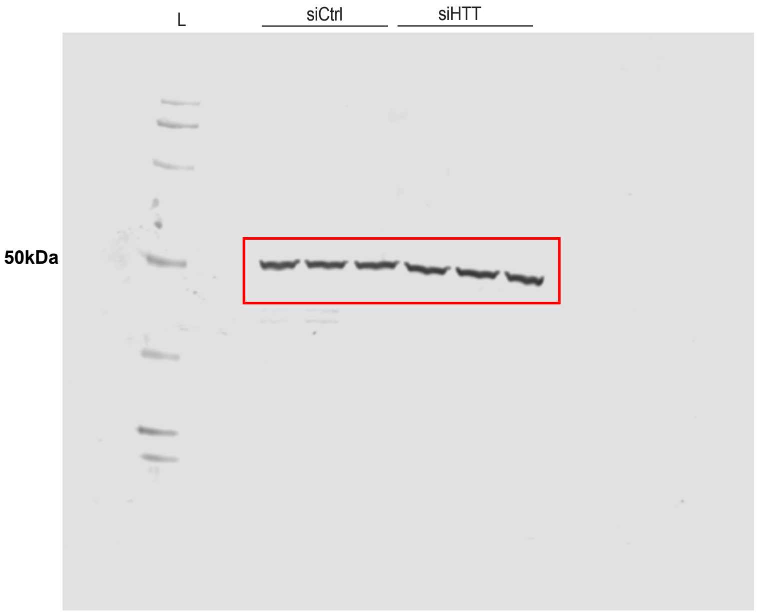

IB: a-tubulin (bw)

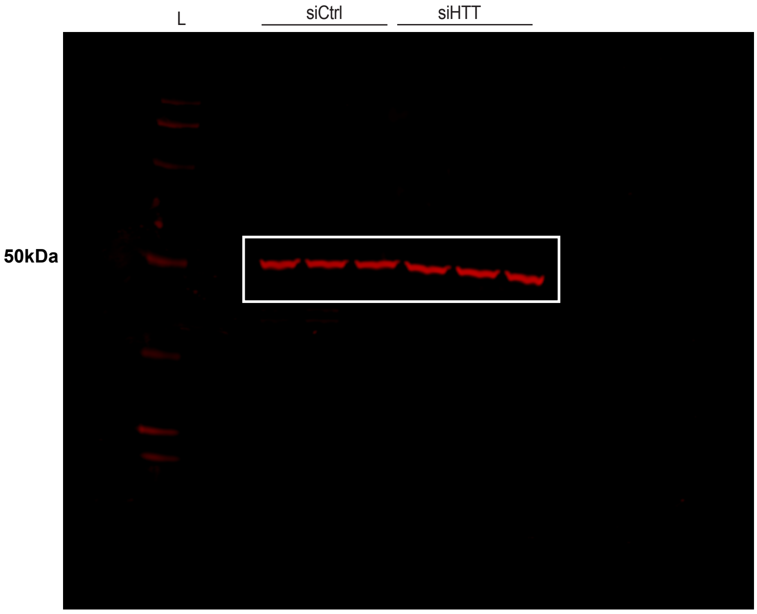

IB: a-tubulin (red)

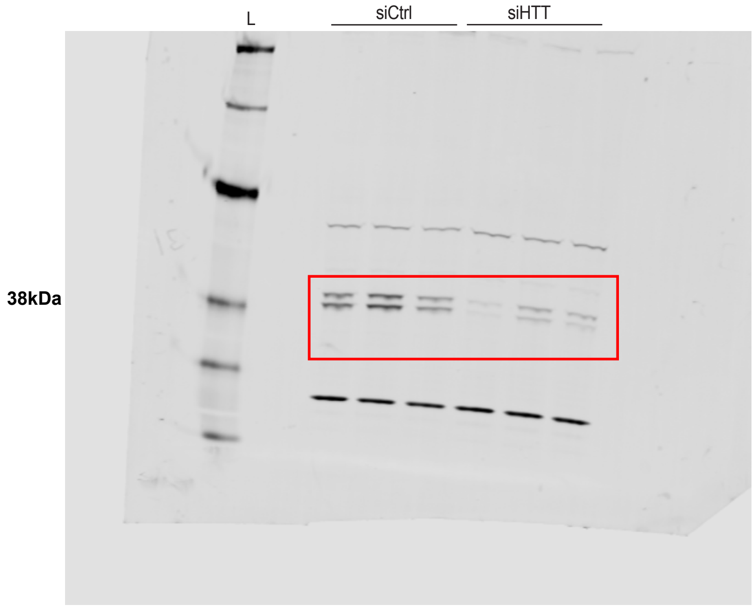

IB: APOE3-mCh-SepH (bw)

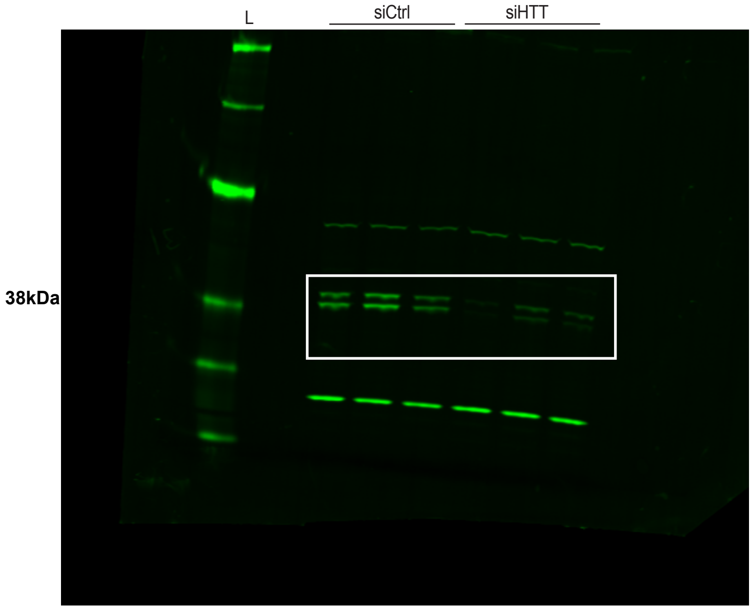

IB: APOE3-mCh-SepH (green)

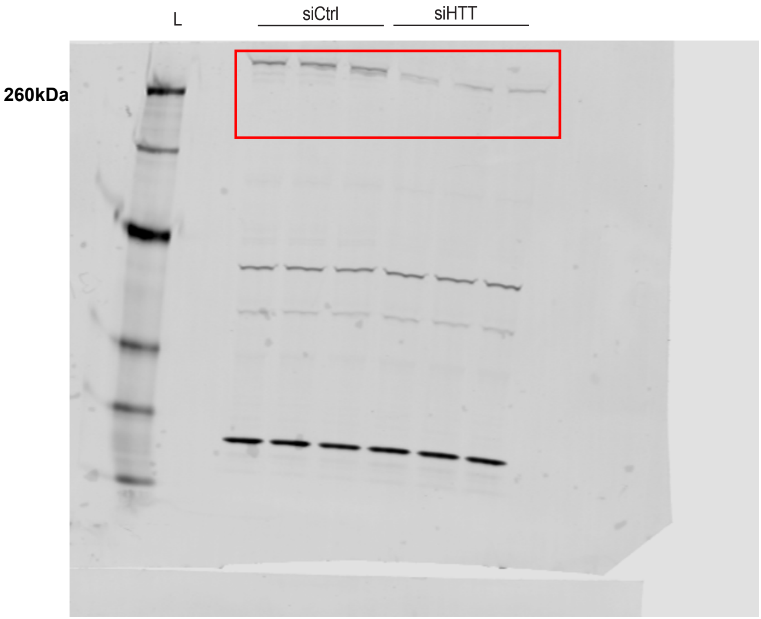

IB: HTT (bw)

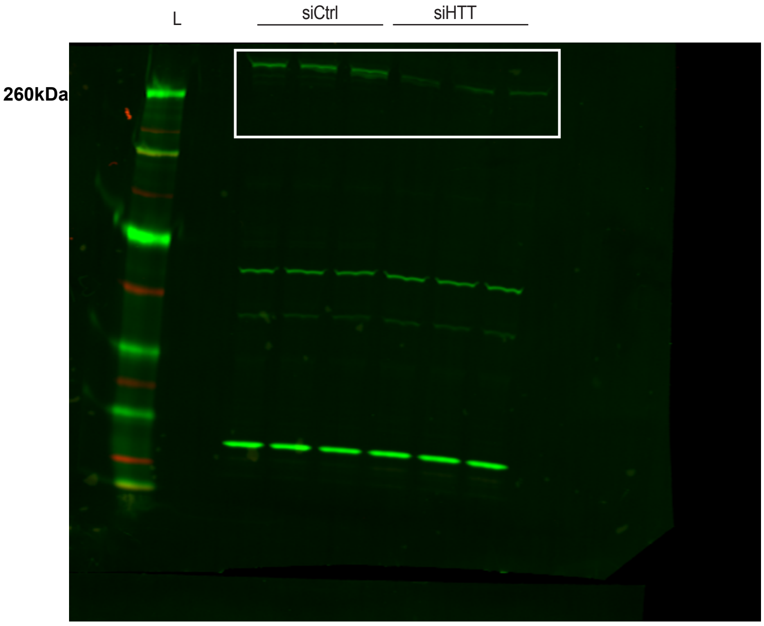

IB: HTT (green)

Uncropped blots for Figure 4B

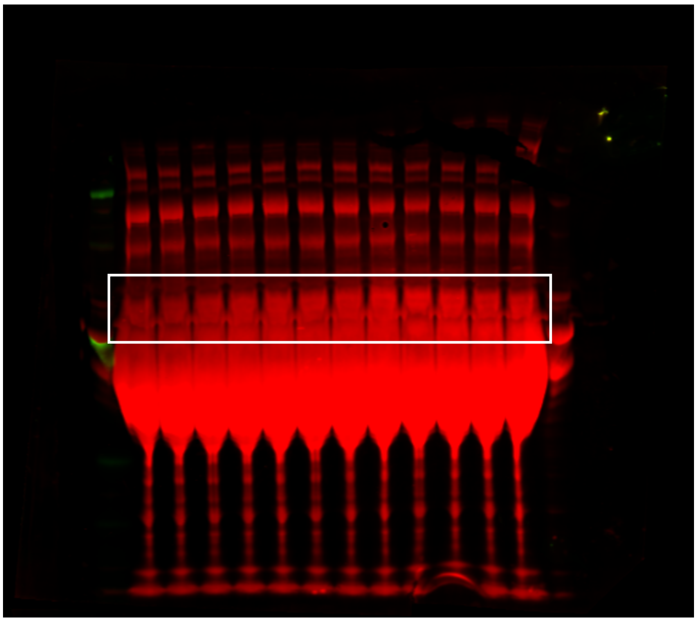

REVERT total protein stain

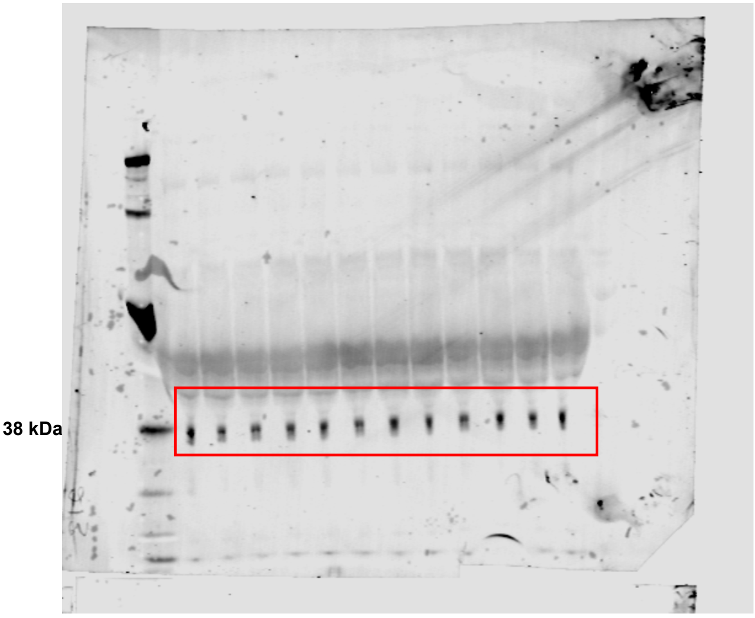

IB: APOE (bw)

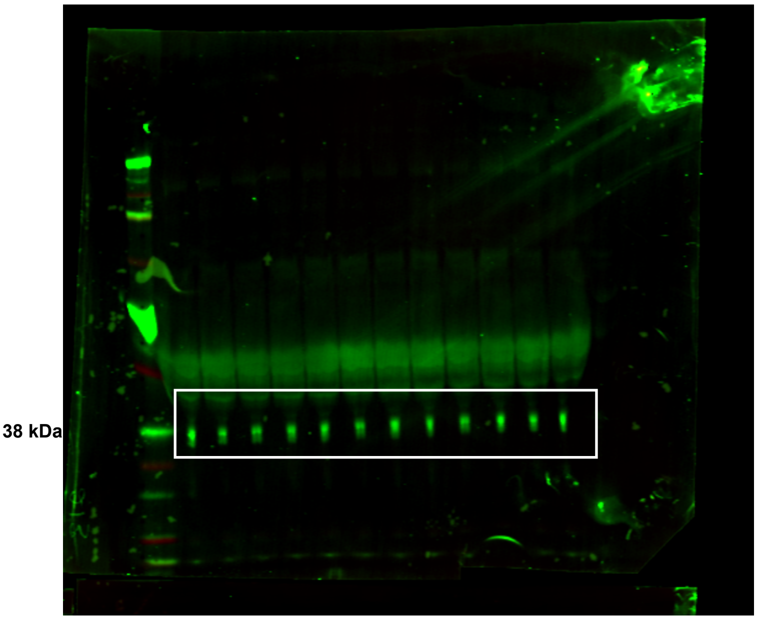

IB: APOE (green)

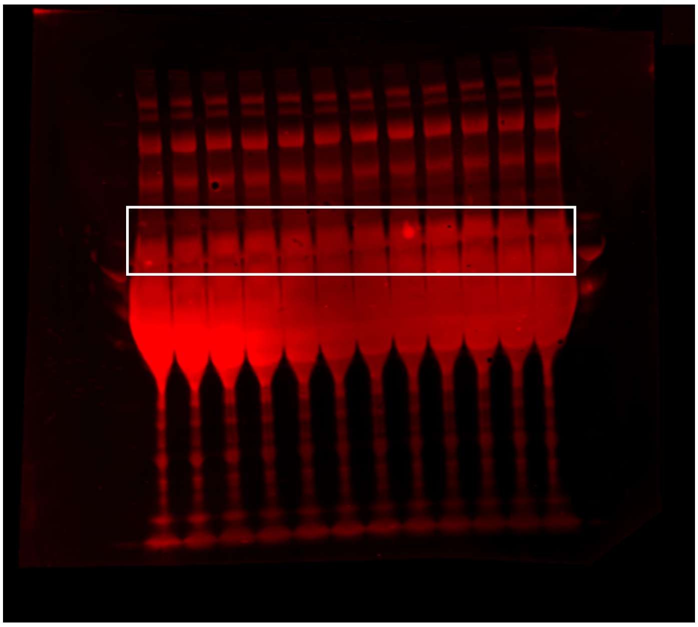

REVERT total protein stain

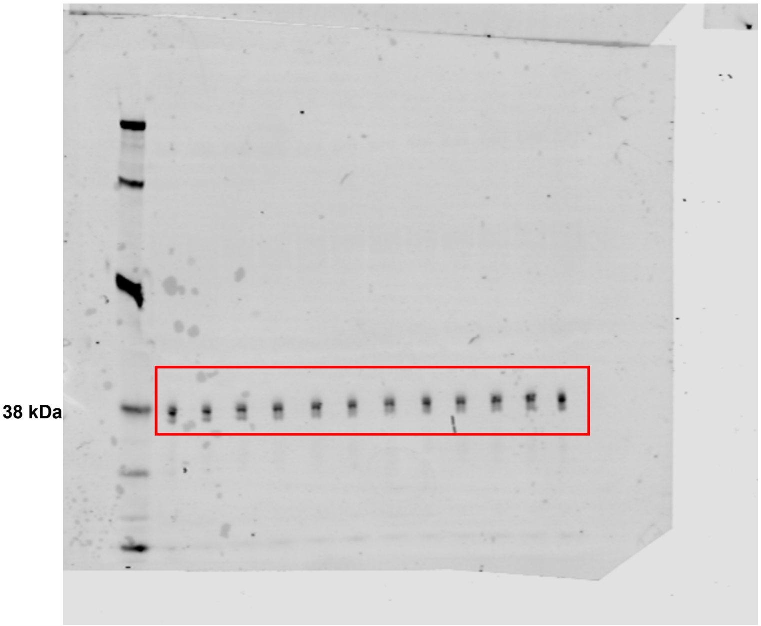

IB: APOE (bw)

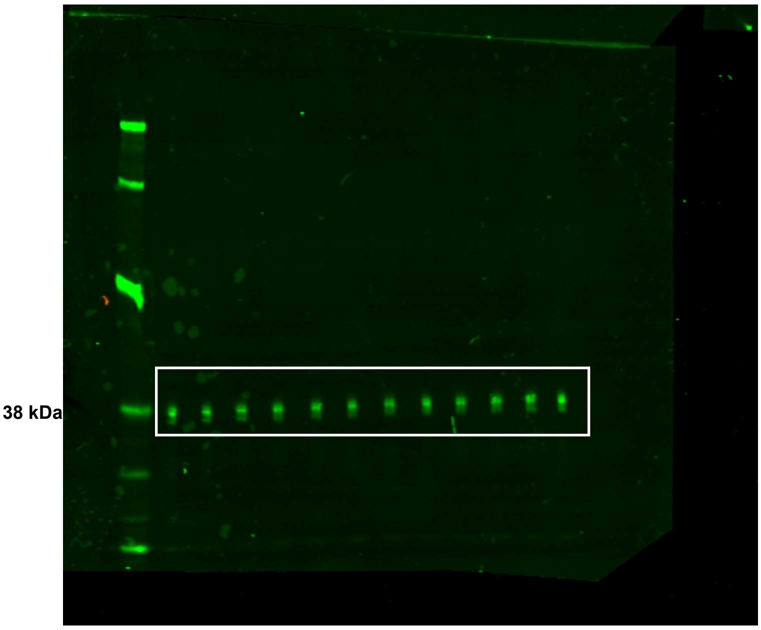

IB: APOE (green)

Uncropped blots for Figure 5A

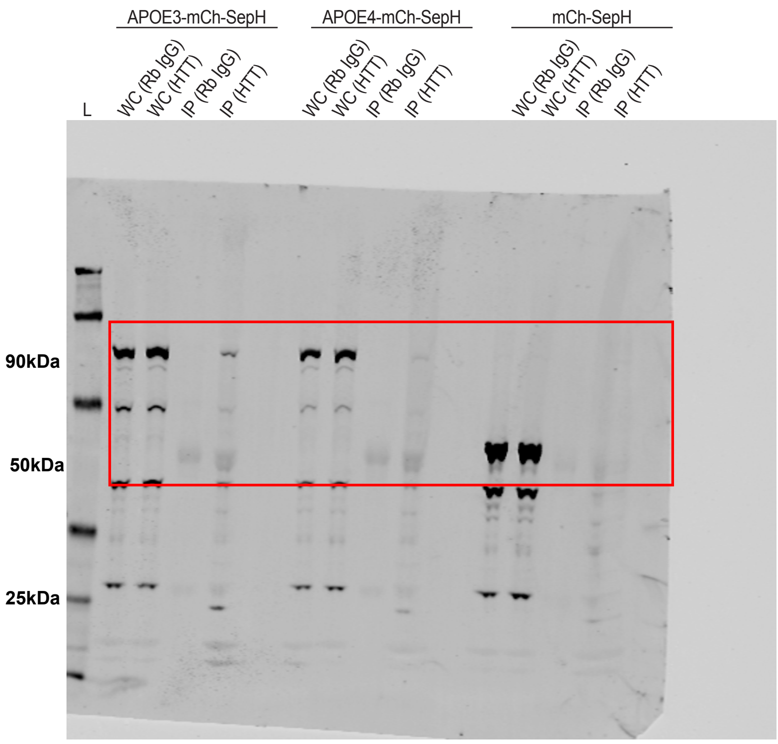

IP: HTT IB: GFP (APOE-mCh-SepH/mCh-Sep) (bw)

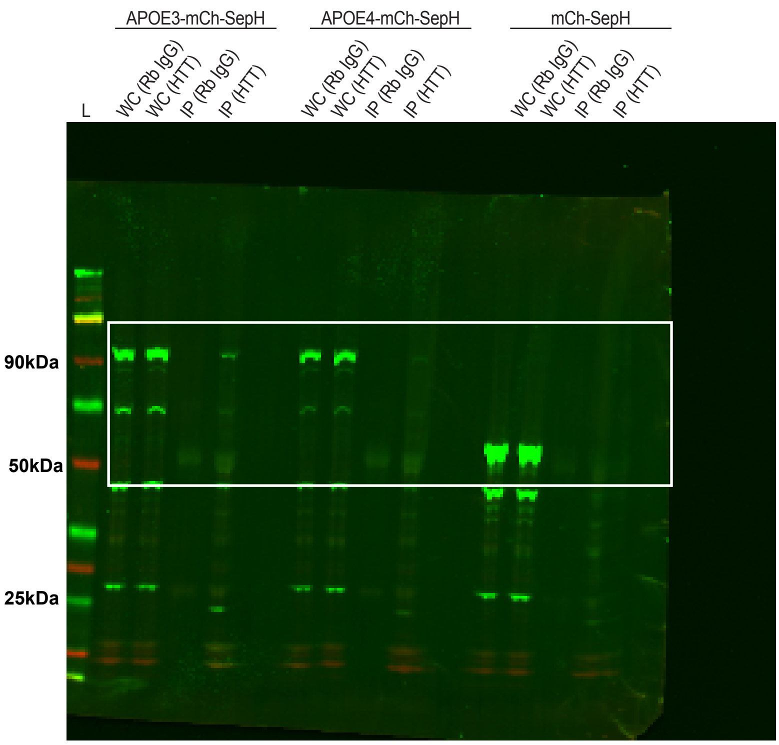

IP: HTT IB: GFP (APOE-mCh-SepH/mCh-Sep)

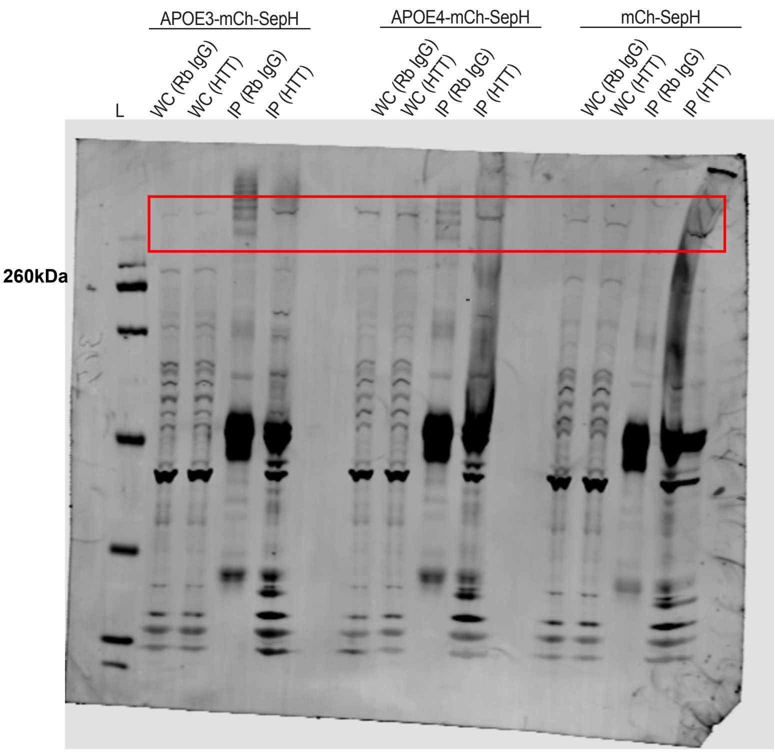

IP: HTT IB: HTT (bw)

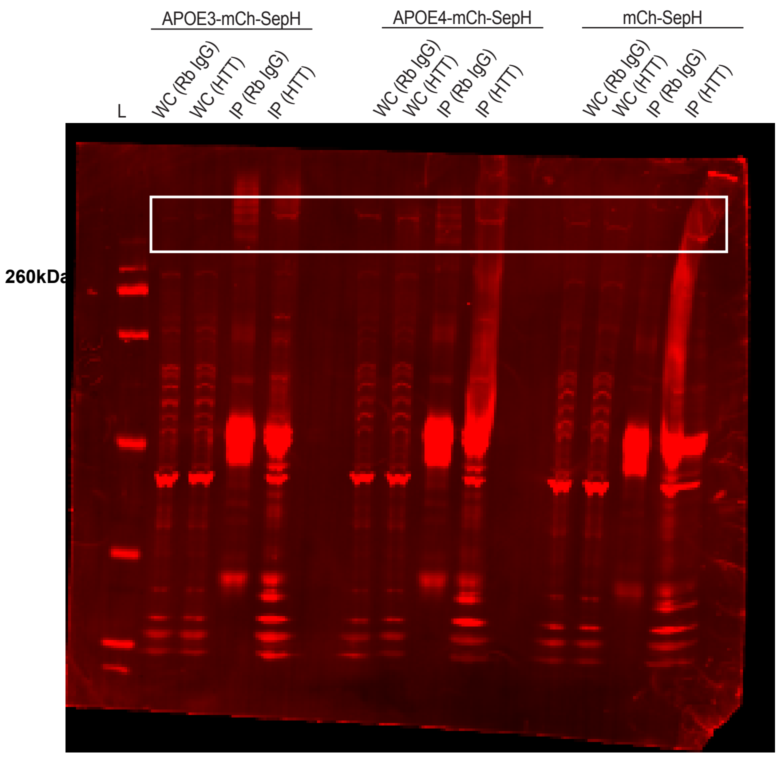

IP: HTT IB: HTT (red)

Uncropped blots for Figure 5B

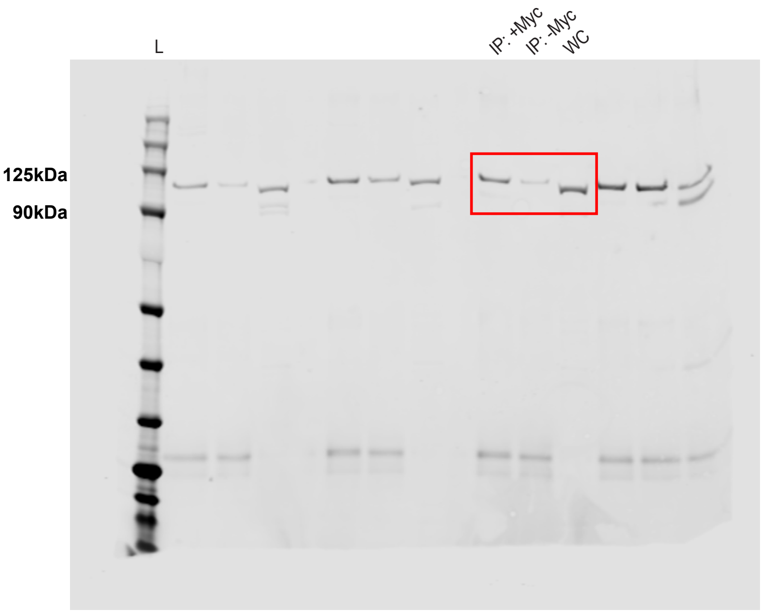

IB: HTT (2416-3144)-Venus

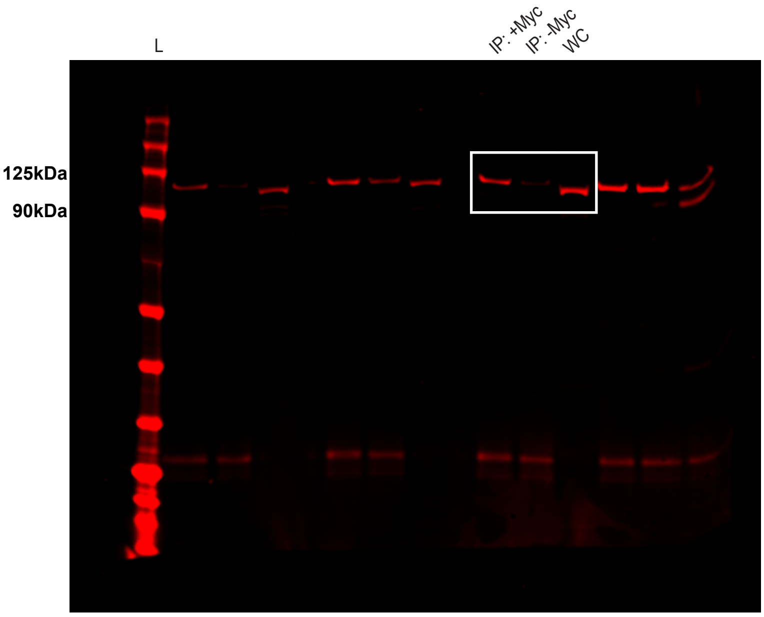

IB: HTT (2416-3144)-Venus

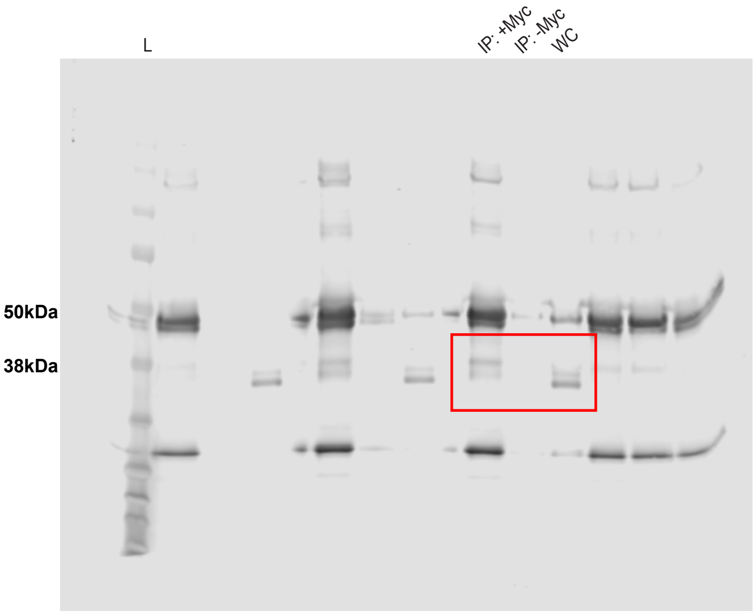

IB: APOE-Myc (bw)

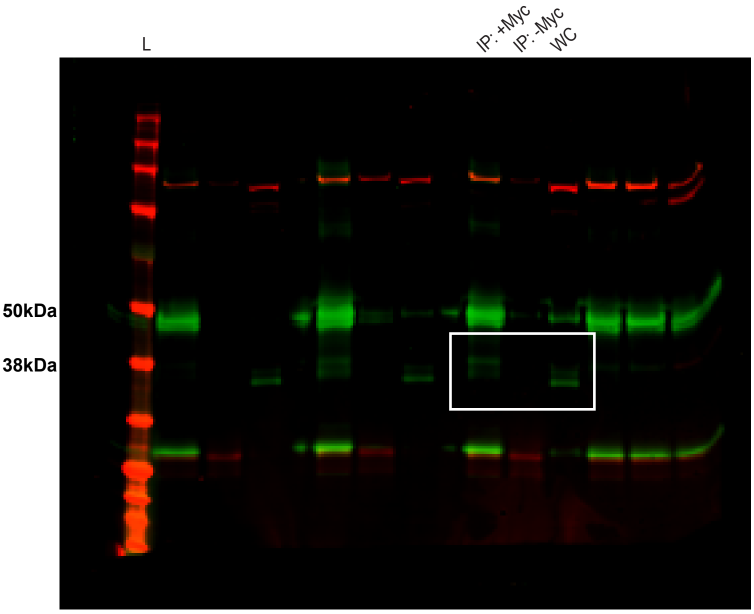

IB: APOE-Myc (green)
